# Primal Subgradient Methods with Predefined Step Sizes

**DOI:** 10.1007/s10957-024-02456-9

**Published:** 2024-05-31

**Authors:** Yurii Nesterov

**Affiliations:** Center for Operations Research and Econometrics (CORE), Catholic University of Louvain (UCL), Ottignies-Louvain-la-Neuve, Belgium

**Keywords:** Convex optimization, Nonsmooth optimization, Subgradient methods, Constrained problems, Optimal Lagrange multipliers

## Abstract

In this paper, we suggest a new framework for analyzing primal subgradient methods for nonsmooth convex optimization problems. We show that the classical step-size rules, based on normalization of subgradient, or on knowledge of the optimal value of the objective function, need corrections when they are applied to optimization problems with constraints. Their proper modifications allow a significant acceleration of these schemes when the objective function has favorable properties (smoothness, strong convexity). We show how the new methods can be used for solving optimization problems with functional constraints with a possibility to approximate the optimal Lagrange multipliers. One of our primal-dual methods works also for unbounded feasible set.

## Introduction

**Motivation.** The first method for unconstrained minimization of nonsmooth convex function was proposed in [8]. This was a primal subgradient method

1.1 $$\begin{aligned} \begin{array}{rcl} x_{k+1}= & {} x_k - h_k s_k, \quad s_k \; = \; f'(x_k), \quad k \ge 0, \end{array} \end{aligned}$$

with constant step sizes $h_k \equiv h > 0$, where $f'(x_k)$ is a subgradient of the objective function at the point $x_k$. In the next years, there were developed several strategies for choosing the steps (see [7] for historical remarks and references). Among them, the most important one is the rule of the *first-order* divergent series:

1.2 $$\begin{aligned} \begin{array}{cc} h_{k} > 0, \quad h_{k} \rightarrow 0, \quad \sum \limits _{k=0}^{\infty } h_{k} = \infty , \end{array} \end{aligned}$$

with the optimal choice $h_k = O(k^{-1/2})$. As a variant, it is possible to use in (2.9) the normalized directions

1.3 $$\begin{aligned} \begin{array}{rcl} s_k= & {} {f'(x_k) \over \Vert f'(x_k) \Vert }. \end{array} \end{aligned}$$

Another alternative for the step sizes is based on the known optimal value $f^*$ [7]:

1.4 $$\begin{aligned} \begin{array}{rcl} h_k= & {} {f(x_k) - f^* \over \Vert f'(x_k) \Vert ^2}. \end{array} \end{aligned}$$

In both cases, the corresponding schemes, as applied to functions with bounded subgradients, have the optimal rate of convergence $O(k^{-1/2})$, established for the best value of the objective function observed during the minimization process [2]. The presence of simple set constraints was treated just by applying to the minimization sequence an Euclidean projection onto the feasible set.

The next important advancement in this area is related to development of the *mirror descent method* (MDM) ( [2], see also [1]). In this scheme, the main information is accumulated in the dual space in the form of aggregated subgradients. For defining the next test point, this object is mapped (*mirrored*) to the primal space by a special prox-function, related to a general norm. Thus, we get an important possibility of describing the topology of convex sets by the appropriate norms.

After this discovery, during several decades, the research activity in this field was concentrated on the development of dual schemes. One of the drawbacks of the classical MDM is that the new subgradients are accumulated with the vanishing weights $h_k$. It was corrected in the framework of *dual averaging* [4], where the aggregation coefficients can be even increasing, and the convergence in the primal space is achieved by applying some vanishing scaling coefficients. Another drawback is related to the fact that the convergence guarantees are traditionally established only for the best values of the objective function. This inconvenience was eliminated by development of *quasi-monotone dual methods* [6], where the rate of convergence is proved for all points of the minimization sequence.

Thus, at some moment, primal methods were almost forgotten. However, in this paper we are going to show that in some situations the primal schemes are very useful. Moreover, there is still space for improvement of the classical methods. Our optimism is supported by the following observations.

Firstly, from the recent developments in Optimization Theory, it becomes clear that the size of subgradients of objective functions for the problems with simple set constraints must be defined differently. Hence, the usual norms in the rules (1.3) and 1.4) can be replaced by more appropriate objects.

Secondly, for an important class of *quasi-convex functions*, linear approximations do not work properly. Hence, for corresponding optimization problems, only the primal schemes can be used. Finally, as we will see, the proper primal schemes provide us with a very simple and natural possibility for approximating the optimal Lagrange multipliers for problems with functional constraints, eliminating somehow the heavy machinery, which is typical for the methods based on Augmented Lagrangian.

**Contents.** In Sect. 2, we present a new subgradient method for minimizing the quasi-convex function on a simple set. Its justification is based on a new concept of *directional proximity measure*, which is a generalization of the old technique initially presented in [3]. In this method, we apply an *indirect strategy* for choosing the step size, which needs a solution of a simple univariate equation. In unconstrained case, this strategy is reduced to the normalization step (1.3). The main advantage of the new method is the possibility of automatic acceleration for functions with Hölder-continuous gradients.

In Sect. 3, we present a method for solving the composite minimization problem with a max-type objective function. For choosing the step size, we use a proper generalization of the rule (1.4), based on an optimal value of the objective. This method admits a linear rate of convergence for smooth strongly convex functions (see Sect. 4). Note that a simple example demonstrates that the classical rule does not benefit from strong convexity. Method of Sect. 3 automatically accelerates on functions with Hölder continuous gradient.

In Sect. 5, we consider a minimization problem with a single max-type constraint containing an additive composite term. For this problem, we present a switching strategy, where the steps for the objective function are based on the rule of Sect. 2, and for improving the feasibility we use the step-size strategy of Sect. 3. For controlling the step sizes, we suggest a new rule of the *second-order divergent series*:

$$\begin{aligned}\begin{array}{rcl} \tau _k\ge & {} \tau _{k+1} \; > \; 0, \quad \sum \limits _{k=0}^{\infty } \tau _k^2 \; = \; \infty . \end{array} \end{aligned}$$

For the bounded feasible sets, it eliminates an unpleasant logarithmic factor in the convergence rate. The method automatically accelerates for the problem with smooth functional components. It is interesting that the rates of convergence for the objective function and the constraints can be different.

The remaining sections of the paper are devoted to the methods, which can approximate optimal Lagrange multipliers for convex problems with functional inequality constraints. In Sect. 6, we consider the simplest switching strategy of this type, where for the steps with the objective function we use the rule of Sect. 2, and for the steps with violated constraints we use the rule of Sect. 3. In the method of Sect. 7, both steps are based on the rule of Sect. 2. In both cases, we obtain the rates of convergence for infeasibility of the generated points, and the upper bound for the duality gap, computed for the simple estimates of the optimal dual multipliers. Such an estimate is formed as a sum of steps at active iterations for each violated constraint divided by the sum of steps at iterations when the objective function was active.

In Sect. 8, we provide the theoretical guarantees for our estimates of the optimal Lagrange multipliers in terms of the value of dual function. They depend on the depth of Slater condition of our problem. Finally, in Sect. 9, we present a switching method, which can generate the approximate dual multipliers for problems with unbounded feasible set.

**Notation.** Denote by ${\mathbb {E}}$ a finite-dimensional real vector space, and by ${\mathbb {E}}^*$ its dual space composed by linear functions on ${\mathbb {E}}$. For such a function $s \in {\mathbb {E}}^*$, denote by $\langle s, x \rangle$ its value at $x \in {\mathbb {E}}$. For measuring distances in ${\mathbb {E}}$, we use an arbitrary norm $\Vert \cdot \Vert$. The corresponding dual norm is defined in a standard way:

$$\begin{aligned} \begin{array}{rcl} \Vert g \Vert _* \; = \; \max \limits _{x \in {\mathbb {E}}} \Big \{ \; \langle g,x \rangle , \; \Vert x \Vert \le 1 \; \Big \}, \quad g \in {\mathbb {E}}^*. \end{array} \end{aligned}$$

Sometimes it is convenient to measure distances in ${\mathbb {E}}$ by Euclidean norm $\Vert \cdot \Vert _B$. It is defined by a self-adjoint positive-definite linear operator $B: {\mathbb {E}}\rightarrow {\mathbb {E}}^*$ in the following way:

1.5 $$\begin{aligned} \begin{array}{rcl} \Vert x \Vert _B= & {} \langle B x, x \rangle ^{1/2}, \; x \in {\mathbb {E}}, \quad \Vert g \Vert ^*_B \; = \; \langle g, B^{-1} g \rangle ^{1/2}, \; g \in {\mathbb {E}}^*. \end{array} \end{aligned}$$

In case of ${\mathbb {E}}= {\mathbb {R}}^n$, for $x \in {\mathbb {R}}^n$, we use the notation $\Vert x \Vert ^2_2 = \sum \limits _{i=1}^n (x^{(i)})^2$.

For a differentiable function $f(\cdot )$ with convex and open domain $\mathrm{dom \,}f \subseteq {\mathbb {E}}$, denote by $\nabla f(x) \in {\mathbb {E}}^*$ its gradient at point $x \in \mathrm{dom \,}f$. If *f* is convex, it can be used for defining the *Bregman distance* between two points $x, y \in \mathrm{dom \,}f$:

1.6 $$\begin{aligned} \begin{array}{rcl} \beta _f(x,y)= & {} f(y) - f(x) - \langle \nabla f(x), y - x \rangle \; \ge \; 0. \end{array} \end{aligned}$$

In this paper, we develop new *proximal-gradient methods* based on a predefined *prox-function* $d(\cdot )$, which can be restricted to a convex open domain $\mathrm{dom \,}d \subseteq {\mathbb {E}}$. This domain always contains the basic feasible set of the corresponding optimization problem. We assume that $d(\cdot )$ is continuously differentiable and *strongly convex* on $\mathrm{dom \,}d$ with parameter one:

1.7 $$\begin{aligned} \begin{array}{rcl} d(y)\ge & {} d(x) + \langle \nabla d(x), y - x \rangle + \hbox { }\ {1 \over 2}\Vert y - x \Vert ^2, \quad x, y \in \mathrm{dom \,}d. \end{array} \end{aligned}$$

Thus, combining the definition (1.6) with inequality (1.7), we get

1.8 $$\begin{aligned} \begin{array}{rcl} \beta _d(x,y)\ge & {} \hbox { }\ {1 \over 2}\Vert x - y \Vert ^2, \quad x, y \in \mathrm{dom \,}d. \end{array} \end{aligned}$$

## Subgradient method for quasi-convex problems

Consider the following constrained optimization problem:

2.1 $$\begin{aligned} \min \limits _{x \in Q} \; f_0(x), \end{aligned}$$

where the function $f_0(\cdot )$ is closed and quasi-convex on $\mathrm{dom \,}f_0$ and the set $Q \subseteq \mathrm{dom \,}f_0$ is closed and convex. Denote by $x^*$ one of the optimal solutions of (2.1) and let $f_0^* = f_0(x^*)$. We assume that

2.2 $$\begin{aligned} \begin{array}{rcl} x^*\in & {} \mathrm{int \,}\mathrm{dom \,}f_0. \end{array} \end{aligned}$$

Let us assume that at any point $x \in Q$ it is possible to compute a vector $f_0'(x) \in {\mathbb {E}}^* \setminus \{0\}$, satisfying the following condition: for any $y \in {\mathbb {E}}$, we have:

2.3 $$\begin{aligned} \begin{array}{rcl} \langle f_0'(x), y - x \rangle \ge 0\Rightarrow & {} f_0(y) \; \ge \; f_0(x). \end{array} \end{aligned}$$

(If $y \not \in \mathrm{dom \,}f_0$, then $f_0(y) {\mathop {=}\limits ^{\textrm{def}}}+ \infty$.) If $f_0(\cdot )$ is differentiable at *x*, then $f'_0(x) = \nabla f_0(x)$.

In order to justify the rate of convergence of our scheme, we need the following characteristic of problem (2.1):

2.4 $$\begin{aligned} \begin{array}{rcl} \mu (r)&{\mathop {=}\limits ^{\textrm{def}}}&\sup \limits _{x \in {\mathbb {E}}} \Big \{ f_0(x) - f_0^*: \; \Vert x - x^* \Vert \le r \Big \}, \quad r \ge 0. \end{array} \end{aligned}$$

If $y \not \in \mathrm{dom \,}f_0$ and $r = \Vert y - x^* \Vert$, then $\mu (r) = + \infty$. In view of assumption (2.2), this function is finite at least in some neighborhood of the origin.

We say that vector $s \in {\mathbb {E}}$ defines a *direction* in ${\mathbb {E}}$ if $\Vert s \Vert = 1$. If $\langle f_0'(x), s \rangle > 0$, we call it a *recession direction* of function $f_0(\cdot )$ at point $x \in Q$. Using such a direction, we can define the *directional proximity measure* of point $x \in Q$ as follows:

2.5 $$\begin{aligned} \begin{array}{rcl} \delta _s(x)&{\mathop {=}\limits ^{\textrm{def}}}&{\langle f_0'(x), x - x^* \rangle \over \langle f_0'(x), s \rangle } \; \ge \; 0. \end{array} \end{aligned}$$

### Lemma 1

Let *s* be a recession direction at point $x \in Q$. Then

2.6 $$\begin{aligned} \begin{array}{rcl} f_0(x) - f_0^*\le & {} \mu (\delta _s(x)). \end{array} \end{aligned}$$

### Proof

Indeed, let us define $y = x^* + \delta _s(x) s$. Then

$$\begin{aligned} \begin{array}{rcl} \langle f_0'(x), y \rangle= & {} \langle f_0'(x), x^* \rangle + \delta _s(x) \langle f_0'(x), s \rangle \; {\mathop {=}\limits ^{(2.5)}} \langle f_0'(x), x \rangle . \end{array} \end{aligned}$$

Since $f_0(\cdot )$ is quasi-convex, this means that $f_0(y) {\mathop {\ge }\limits ^{(2.3)}} f_0(x)$. Therefore,

$$\begin{aligned} \begin{array}{rcl} f_0(x) - f_0^*\le & {} f_0(y) - f_0^* \; {\mathop {\le }\limits ^{(2.4)}} \; \mu (\Vert y - x^* \Vert ) \; = \; \mu (\delta _s(x)). \end{array} \end{aligned}$$

$$\square $$

In our analysis, we use the following univariate functions:

2.7 $$\begin{aligned} \begin{array}{rcl} \varphi _{{{\bar{x}}}}(\lambda )= & {} \max \limits _{x \in Q} \Big \{ \lambda \langle f_0'({{\bar{x}}}), {{\bar{x}}} - x \rangle - \beta _d({{\bar{x}}}, x) \Big \}, \quad \lambda \ge 0, \end{array} \end{aligned}$$

where ${{\bar{x}}} \in Q$. The optimal solution of this optimization problem is denoted by $T_{{{\bar{x}}}}(\lambda )$. Note that function $\varphi _{{{\bar{x}}}}(\cdot )$ is convex and continuously differentiable with the following derivative:

2.8 $$\begin{aligned} \begin{array}{rcl} \varphi _{{{\bar{x}}}}'(\lambda )= & {} \langle f_0'({{\bar{x}}}), {{\bar{x}}} - T_{\bar{x}}(\lambda ) \rangle . \end{array} \end{aligned}$$

Thus, $\varphi _{{{\bar{x}}}}(0) = 0$, $T_{{{\bar{x}}}}(0) = {{\bar{x}}}$, and $\varphi '_{{{\bar{x}}}}(0) = 0$. Consequently, $\varphi _{{{\bar{x}}}}(\lambda ) \ge 0$ for all $\lambda \ge 0$.

The basic subgradient method for solving the problem (2.1) looks as follows.2.9

### Lemma 2

At each iteration $k \ge 0$ of method (2.9), for any $x \in Q$, we have

2.10 $$\begin{aligned} \begin{array}{rcl} \beta _d(x_{k+1},x)\le & {} \beta _d(x_k,x ) - \lambda _k \langle f_0'(x_k), x_k - x \rangle + \varphi _{x_k}(\lambda _k). \end{array} \end{aligned}$$

### Proof

Note that the first-order optimality condition for the problem at Step b) can be written as follows:

2.11 $$\begin{aligned} \begin{array}{rcl} \langle \lambda _k f_0'(x_k) + \nabla d(x_{k+1}) - \nabla d(x_k), x - x_{k+1} \rangle\ge & {} 0, \quad \forall x \in Q. \end{array} \end{aligned}$$

Therefore, we have

$$\begin{aligned} \begin{array}{rl} &{} \beta _d(x_{k+1},x) \; {\mathop {=}\limits ^{(1.6)}} \; d(x) - d(x_{k+1}) - \langle \nabla d(x_{k+1}), x - x_{k+1} \rangle \\ \\ &{}\quad = \beta _d(x_{k},x) + d(x_{k}) + \langle \nabla d(x_{k}), x - x_k \rangle - d(x_{k+1}) - \langle \nabla d(x_{k+1}), x - x_{k+1} \rangle \\ \\ &{}\quad {\mathop {\le }\limits ^{(2.11)}} \beta _d(x_{k},x) + d(x_k) + \langle \nabla d(x_{k}), x - x_k \rangle - d(x_{k+1}) \\ \\ &{}\qquad + \lambda _k \langle f'_0(x_k), x - x_{k+1} \rangle - \langle \nabla d(x_k), x - x_{k+1} \rangle \\ \\ &{}\quad = \beta _d(x_{k},x) + \lambda _k \langle f'_0(x_k), x - x_{k+1} \rangle - \beta _d(x_k,x_{k+1})\\ \\ &{}\quad = \beta _d(x_{k},x) - \lambda _k \langle f'_0(x_k), x_k - x \rangle + \varphi _{x_k}(\lambda _k). \end{array} \end{aligned}$$

$$\square $$

In our method, we will use an *indirect control* of the *dual* step sizes $\{ \lambda _k \}_{k\ge 0}$, which is based on a predefined sequence of *primal* step-size parameters $\{ h_k \}_{k\ge 0}$. As we will prove by Lemma 3, the convergence of the process can be derived, for example, from the following standard conditions:

2.12 $$\begin{aligned} \begin{array}{rcl} \{ h_k \}_{k \ge 0}: \quad \hbox {a) }h_k > 0, \quad \hbox {b) }\sum \limits _{k=0}^{\infty } h_k = \infty . \end{array} \end{aligned}$$

Then, inequality (2.10) justifies the choice of the *dual* step-size parameter $\lambda _k$ as a solution to the following equation:2.13

### Example 1

If $Q = {\mathbb {E}}$ and $d(x) = \hbox { }\ {1 \over 2}\Vert x \Vert ^2_B$, then $\varphi _{{{\bar{x}}}}(\lambda ) {\mathop {=}\limits ^{(2.7)}} {\lambda ^2 \over 2} (\Vert f'_0({{\bar{x}}}) \Vert ^*_B)^2$, and equation (2.13) gives us $\lambda _k = h_k /\Vert f'_0(x_k) \Vert ^*_B$. In this case, method (2.9) coincides with the classical variant of subgradient method $x_{k+1} = x_k - h_k {f'_0(x_k) \over \Vert f'_0(x_k) \Vert ^*_B}$. However, for $Q \ne {\mathbb {E}}$, the rule (2.13) allows a proper scaling of the step size by the boundary of feasible set. $\square$

At each iteration of method (2.9), the corresponding value of $\lambda _k$ can be found by an efficient one-dimensional search procedure, based, for example, on a Newton-type scheme. Since the latter scheme has local quadratic convergence, we make a plausible assumption that it is possible to compute an exact solution of the equation (2.13). This solution has the following important interpretation (in view of (2.8)):

2.14 $$\begin{aligned} \begin{array}{rcl} \lambda _k= & {} \max \limits _{\lambda \ge 0} \Big \{ \lambda : \; \varphi _{x_k}(\lambda ) \le \hbox { }\ {1 \over 2}h_k^2 \Big \}. \end{array} \end{aligned}$$

At the same time, we have $\langle \lambda _k f_0'(x_k) + \nabla d(x_{k+1}) - \nabla d(x_k), x_k - x_{k+1} \rangle {\mathop {\ge }\limits ^{(2.11)}} 0$. Thus

$$\begin{aligned} \begin{array}{rcl} \lambda _k \langle f'_0(x_k), x_k - x_{k+1} \rangle &{} {\mathop {\ge }\limits ^{(1.6)}} &{} \beta _d(x_k,x_{k+1}) + \beta _d(x_{k+1},x_k) \\ \\ &{} {\mathop {\ge }\limits ^{(1.8)}} &{} \beta _d(x_k,x_{k+1}) + \hbox { }\ {1 \over 2}\Vert x_k - x_{k+1} \Vert ^2. \end{array} \end{aligned}$$

Hence

2.15 $$\begin{aligned} \begin{array}{rcl} \hbox { }\ {1 \over 2}h_k^2&{\mathop {=}\limits ^{(2.13)}}&\varphi _{x_k}(\lambda _k) \; {\mathop {\ge }\limits ^{(2.7)}} \; \hbox { }\ {1 \over 2}\Vert x_k - x_{k+1} \Vert ^2, \quad k \ge 0. \end{array} \end{aligned}$$

Our complexity bounds follow from the rate of convergence of the following values:

$$\begin{aligned} \begin{array}{rcl} \delta _k&{\mathop {=}\limits ^{\textrm{def}}}&\delta _{s_k}(x_k), \quad s_k \; {\mathop {=}\limits ^{\textrm{def}}}\; {x_k - x_{k+1} \over \Vert x_k - x_{k+1} \Vert }. \end{array} \end{aligned}$$

### Lemma 3

Let condition (2.13) be satisfied. Then for any $N \ge 0$, we have

2.16 $$\begin{aligned} \begin{array}{rcl} \sum \limits _{k=0}^N h_k \delta _k\le & {} \beta _d(x_0,x^*) + \hbox { }\ {1 \over 2}\sum \limits _{k=0}^N h_k^2. \end{array} \end{aligned}$$

### Proof

Indeed, in view of inequality (2.10), for $r_k = \beta _d(x_k,x^*)$, we have

$$\begin{aligned} \begin{array}{rcl} r_{k+1}\le & {} r_k - \lambda _k \langle f_0'(x_k), x_k - x^* \rangle + \varphi _{x_k}(\lambda _k) \; {\mathop {=}\limits ^{(2.13)}} \; r_k - \lambda _k \langle f_0'(x_k), x_k - x^* \rangle + \hbox { }\ {1 \over 2}h_k^2. \end{array} \end{aligned}$$

On the other hand,

$$\begin{aligned} \begin{array}{rcl} \lambda _k \langle f_0'(x_k), x_k - x_{k+1} \rangle &{} {\mathop {=}\limits ^{(2.13)}} &{} \beta _d(x_k,x_{k+1}) + \hbox { }\ {1 \over 2}h_k^2 \; {\mathop {\ge }\limits ^{(1.8)}} \; \hbox { }\ {1 \over 2}\Vert x_k - x_{k+1} \Vert ^2 + \hbox { }\ {1 \over 2}h_k^2 \\ \\ &{} \ge &{} h_k \Vert x_k - x_{k+1} \Vert . \end{array} \end{aligned}$$

Hence,

2.17 $$\begin{aligned} \begin{array}{rcl} r_{k+1}\le & {} r_k - h_k \delta _k + \hbox { }\ {1 \over 2}h_k^2. \end{array} \end{aligned}$$

Summing up these inequalities for $k = 0, \dots , N$, we obtain inequality (2.16). $\square$

Let us look now at one example of the rate of convergence of method (2.9) for an objective function from a nonstandard problem class. For simplicity, let us measure distances by Euclidean norm $\Vert x \Vert _B$ (see (1.5)). In this case, we can take $d(x) = \hbox { }\ {1 \over 2}\Vert x \Vert ^2_B$ and get $\beta _d(x,y) = \hbox { }\ {1 \over 2}\Vert x - y \Vert ^2_B$. Let us assume that the function $f_0(\cdot )$ in problem (2.1) is *p*-times continuously differentiable on ${\mathbb {E}}$ and its *p*th derivative is Lipschitz-continuous with constant $L_p$. Then we can bound the function $\mu (\cdot )$ in (2.4) as follows:

2.18 $$\begin{aligned} \begin{array}{rcl} \mu (r)\le & {} \sum \limits _{i=1}^p {r^i \over i!} \Vert D^i f_0(x^*) \Vert + {L_p \over (p+1)!} r^{p+1}, \quad r \ge 0, \end{array} \end{aligned}$$

where all norms for derivatives are induced by $\Vert \cdot \Vert _B$.

Let us fix the total number of steps $N \ge 1$ and assume the bound $R_0 \ge \Vert x_0 - x^* \Vert _B$ be available. Then, defining the step sizes

2.19 $$\begin{aligned} \begin{array}{rcl} h_k= & {} h \; {\mathop {=}\limits ^{\textrm{def}}}\; {R_0 \over \sqrt{N+1}}, \quad 0 \le k \le N, \end{array} \end{aligned}$$

we get $\delta ^*_N = \min \limits _{0 \le k \le N} \delta _k \; {\mathop {\le }\limits ^{(2.16)}}{R_0 \over \sqrt{N+1}}$. Hence, in view of inequality (2.18), we have

2.20 $$\begin{aligned} \begin{array}{rcl} f_N^*&{\mathop {=}\limits ^{\textrm{def}}}&\min \limits _{0 \le k \le N} f(x_k) \; \le \; f_0^* + \sum \limits _{i=1}^p {1 \over i!} \Vert D^i f_0(x^*) \Vert \left( {R_0 \over \sqrt{N+1}} \right) ^i + {L_p \over (p+1)!} \left( {R_0 \over \sqrt{N+1}} \right) ^{p+1}. \end{array}\nonumber \\ \end{aligned}$$

Note that the first *p* coefficients in estimate (2.20) depend on the local properties of the objective function at the solution, and only the last term employs the global Lipschitz constant for the *p*th derivative. Clearly, we do not need to know the bounds for all these derivatives in order to define the step-size strategy (2.19).

## Step-size control for max-type convex problems

Let us consider now the following problem of *composite optimization*

3.1 $$\begin{aligned} \begin{array}{rcl} F_* \; {\mathop {=}\limits ^{\textrm{def}}}\; \min \limits _{x \in \mathrm{dom \,}\psi } \Big \{ \; F(x)= & {} f(x) + \psi (x) \; \Big \}, \end{array} \end{aligned}$$

where $\psi (\cdot )$ is a simple closed convex function, and

3.2 $$\begin{aligned} \begin{array}{rcl} f(x)= & {} \max \limits _{1 \le i \le m} \; f_i(x), \quad x \in \mathrm{dom \,}\psi , \end{array} \end{aligned}$$

with all $f_i(\cdot )$, $1 \le i \le m$, being closed and convex on $\mathrm{dom \,}\psi$. Denote by

$$\begin{aligned} \begin{array}{rcl} \ell _{{{\bar{x}}}}(x)= & {} \max \limits _{1 \le i \le m} [ \; f_i(\bar{x}) + \langle f'_i({{\bar{x}}}), x - {{\bar{x}}} \rangle \, ] \; \le \; f(x), \quad x \in \mathrm{dom \,}\psi , \end{array} \end{aligned}$$

the linearization of function $f(\cdot )$, and by $x_* \in \mathrm{dom \,}\psi$ an optimal solution of this problem.

Similarly to (2.7), let us define the following univariate functions:

3.3 $$\begin{aligned} \begin{array}{rcl} {{\hat{\varphi }}}_{{{\bar{x}}}}(\lambda )= & {} \max \limits _{x \in \mathrm{dom \,}\psi } \Big \{ \lambda \Big [ F({{\bar{x}}}) - \ell _{{{\bar{x}}}}(x) - \psi (x) \Big ] - \beta _d({{\bar{x}}}, x) \Big \}, \quad \lambda \ge 0, \end{array} \end{aligned}$$

where ${{\bar{x}}} \in \mathrm{dom \,}\psi$. The unique optimal solution of this optimization problem is denoted by ${{\hat{T}}}_{{{\bar{x}}}}(\lambda )$. Note that function ${{\hat{\varphi }}}_{{{\bar{x}}}}(\cdot )$ is convex and continuously differentiable with the following derivative:

3.4 $$\begin{aligned} \begin{array}{rcl} {{\hat{\varphi }}}_{{{\bar{x}}}}'(\lambda )= & {} - \Big [ \ell _{{{\bar{x}}}}({{\hat{T}}}) + \psi ({{\hat{T}}}) - F({{\bar{x}}})\Big ], \quad \lambda \ge 0, \end{array} \end{aligned}$$

where ${{\hat{T}}} = {{\hat{T}}}_{{{\bar{x}}}}(\lambda )$. Thus, ${{\hat{\varphi }}}_{\bar{x}}(0) = 0$, ${{\hat{T}}}_{{{\bar{x}}}}(0) = {{\bar{x}}}$, and ${{\hat{\varphi }}}'_{\bar{x}}(0) = 0$. Hence, ${{\hat{\varphi }}}_{{{\bar{x}}}}(\lambda ) \ge 0$ for all $\lambda \ge 0$. Let us prove the following variant of Lemma 2.

### Lemma 4

Let ${{\bar{x}}} \in \mathrm{dom \,}\psi$ and ${{\hat{T}}} = {{\hat{T}}}_{{{\bar{x}}}}(\lambda )$ for some $\lambda \ge 0$. Then

3.5 $$\begin{aligned} \begin{array}{rcl} \beta _d({{\hat{T}}},x_*)\le & {} \beta _d({{\bar{x}}}, x_*) + \lambda \Big [ \ell _{{{\bar{x}}}}(x_*) + \psi (x_*) - F({{\bar{x}}}) \Big ] + {{\hat{\varphi }}}_{\bar{x}}(\lambda ). \end{array} \end{aligned}$$

### Proof

In view of the first-order optimality condition for the minimization problem in (3.3), for all $x \in \mathrm{dom \,}\psi$, we have

3.6 $$\begin{aligned} \begin{array}{rcl} \langle \nabla d({{\hat{T}}}) - \nabla d({{\bar{x}}}), x - {{\hat{T}}} \rangle + \lambda \Big [ \ell _{{{\bar{x}}}}(x)+ \psi (x) \Big ]\ge & {} \lambda \Big [ \ell _{{{\bar{x}}}}({{\hat{T}}})+ \psi ({{\hat{T}}}) \Big ]. \end{array} \end{aligned}$$

Therefore, we get

$$\begin{aligned} \begin{array}{rcl} \beta _d({{\hat{T}}}, x_*) &{} = &{} \beta _d({{\bar{x}}}, x_*) + d({{\bar{x}}}) + \langle \nabla d({{\bar{x}}}), x_* - {{\bar{x}}} \rangle - d({{\hat{T}}}) - \langle \nabla d({{\hat{T}}}), x^* - {{\hat{T}}} \rangle \\ \\ &{} {\mathop {\le }\limits ^{(3.6)}} &{} \beta _d({{\bar{x}}}, x_*) + d({{\bar{x}}}) + \langle \nabla d({{\bar{x}}}), x_* - {{\bar{x}}} \rangle - d({{\hat{T}}}) \\ \\ &{} &{} - \langle \nabla d({{\bar{x}}}), x_* - {{\hat{T}}} \rangle + \lambda \Big [ \ell _{{{\bar{x}}}}(x_*)+ \psi (x_*) - \ell _{{{\bar{x}}}}({{\hat{T}}}) - \psi ({{\hat{T}}}) \Big ]\\ \\ &{} {\mathop {=}\limits ^{(3.3)}} &{} \beta _d({{\bar{x}}}, x_*) + \lambda \Big [ \ell _{\bar{x}}(x_*)+ \psi (x_*) - F({{\bar{x}}}) \Big ] + {{\hat{\varphi }}}_{{{\bar{x}}}}(\lambda ). \end{array} \end{aligned}$$

$$\square $$

In this section, we analyze the following optimization scheme.3.7Suppose that the optimal value $F_*$ is known. In view of convexity of the function $f(\cdot )$, we have

$$\begin{aligned} \begin{array}{rcl} \ell _{{{\bar{x}}}}(x_*) + \psi (x_*) - F({{\bar{x}}})\le & {} F_* - F({{\bar{x}}}). \end{array} \end{aligned}$$

Hence, for points $\{ x_k \}_{k \ge 0}$, generated by method (3.7), we have

3.8 $$\begin{aligned} \begin{array}{rcl} \beta _d(x_{k+1},x_*)&{\mathop {\le }\limits ^{(3.5)}}&\beta _d(x_k,x_*) + \Big ({{\hat{\varphi }}}_{x_k}(\lambda _k) - \lambda _k [F(x_k) - F_*] \Big ). \end{array} \end{aligned}$$

This observation explains the following step-size strategy:3.9

This strategy has a natural optimization interpretation.

### Lemma 5

Let $\lambda _k$ be defined by (3.9). Then

3.10 $$\begin{aligned} \begin{array}{rcl} {{\hat{T}}}_{x_k}(\lambda _k)= & {} \arg \min \limits _{x \in \mathrm{dom \,}\psi } \Big \{ \;\beta _d(x_k,x): \; \ell _{x_k}(x) + \psi (x) \le F_* \; \Big \}. \end{array} \end{aligned}$$

### Proof

Indeed,

$$\begin{aligned} \begin{array}{rl} &{} \min \limits _{\lambda \ge 0} \Big \{ \; {{\hat{\varphi }}}_{x_k}(\lambda ) - \lambda [F(x_k) - F_*] \; \Big \} \\ \\ &{}\quad {\mathop {=}\limits ^{(3.3)}} \min \limits _{\lambda \ge 0} \max \limits _{x \in \mathrm{dom \,}\psi } \Big \{ \; - \lambda \Big [ \ell _{x_k}(x) + \psi (x) -F(x_k) \Big ] - \beta _d(x_k,x) - \lambda [F(x_k) - F_*] \; \Big \}\\ \\ &{}\quad = \max \limits _{x \in \mathrm{dom \,}\psi } \min \limits _{\lambda \ge 0} \Big \{ \; - \beta _d(x_k,x) + \lambda \Big [ F_* - \ell _{x_k}(x) - \psi (x) ] \Big ] \; \Big \}\\ \\ &{}\quad = - \min \limits _{x \in \mathrm{dom \,}\psi }\Big \{ \; \beta _d(x_k,x): \; \ell _{x_k}(x) + \psi (x) \le F_* \; \Big \}. \end{array} \end{aligned}$$

$$\square $$

Lemma 5 has two important consequences allowing us to estimate the rate of convergence of method (3.7) with the step-size rule (3.9). Namely, for any $k \ge 0$ we have

3.11 $$\begin{aligned} \begin{array}{rcl} \beta _d(x_{k+1},x_*)&{\mathop {\le }\limits ^{(3.8)}}&\beta _d(x_k,x_*) - \beta _d(x_k,x_{k+1}), \end{array} \end{aligned}$$

3.12 $$\begin{aligned} \begin{array}{rcl} \ell _{x_k}(x_{k+1}) + \psi (x_{k+1})&{\mathop {\le }\limits ^{(3.10)}} F_*. \end{array} \end{aligned}$$

Note that method (3.7) is not monotone. However, inequality (3.11) gives us a global rate of convergence for the following characteristic:

3.13 $$\begin{aligned} \begin{array}{rcl} \rho _k&{\mathop {=}\limits ^{\textrm{def}}}&\min \limits _{0 \le i \le k} \Vert x_i - x_{i+1} \Vert \; {\mathop {\le }\limits ^{(1.8)}} \; \min \limits _{0 \le i \le k} \sqrt{ 2 \beta _d(x_i,x_{i+1})} \; {\mathop {\le }\limits ^{(3.11)}} \; {r_0 \over \sqrt{k+1}}, \quad k \ge 0, \end{array}\nonumber \\ \end{aligned}$$

where $$r_0 = \beta _d(x_0,x_*)$$.

### Theorem 1

Let all functions $f_i(\cdot )$ have Hölder-continuous gradients:

3.14 $$\begin{aligned} \begin{array}{rcl} f_i(y)\le & {} f_i(x) + \langle f'_i(x), y - x \rangle + {L_{\nu } \over 1 + \nu } \Vert y - x \Vert ^{1+\nu }, \quad x, y \in \mathrm{dom \,}\psi , \; 1 \le i \le p, \end{array}\nonumber \\ \end{aligned}$$

where $\nu \in [0,1]$ and $L_{\nu } \ge 0$. Then for the step-size rule (3.9) in method (3.7) we have

3.15 $$\begin{aligned} \begin{array}{rcl} F_{k}&{\mathop {=}\limits ^{\textrm{def}}}&\min \limits _{0 \le i \le k} F(x_i) \; \le \; F_* + {L_{\nu } r_0^{1+\nu } \over 1+\nu } \left( {1 \over k} \right) ^{1 + \nu \over 2}, \quad k \ge 1. \end{array} \end{aligned}$$

### Proof

Indeed, let $\rho _k = \Vert x_{i_k} - x_{i_k+1} \Vert$ for some $i_k$, $0 \le i_k \le k$. Then for any $k \ge 0$ we have

$$\begin{aligned} \begin{array}{rcl} F_{k+1} \; \le \; F(x_{i_k + 1}) &{} {\mathop {\le }\limits ^{(3.14)}} &{} \ell _{x_{i_k}}(x_{i_k + 1}) + {L_{\nu } \over 1 + \nu } \rho _k^{1+\nu } + \psi (x_{i_k+1})\\ \\ &{} {\mathop {\le }\limits ^{(3.12)}} &{} F_* + {L_{\nu } \over 1 + \nu } \rho _k^{1+\nu }. \end{array} \end{aligned}$$

It remains to use inequality (3.13). $\square$

Note that the step-size strategy (3.9) does not depend on the Hölder parameter $\nu$ in the condition (3.14). Hence, the number of iterations of method (3.7), (3.9), ensuring an $\epsilon$-accuracy in function value, is bounded from above by the following quantity:

3.16 $$\begin{aligned} \begin{array}{c} \inf \limits _{0 \le \nu \le 1} \left[ {L_{\nu } \over (1+\nu ) \epsilon } \right] ^{2 \over 1 + \nu } \, r_0^2. \end{array} \end{aligned}$$

To conclude the section, let us show that the step-size rule (3.9) can behave much better than the classical one.

### Example 2

Let ${\mathbb {E}}= {\mathbb {R}}^2$, $\Vert x \Vert ^2 = x^T x$, and $f(x) = \hbox { }\ {1 \over 2}(x^{(1)})^2 + \hbox { }\ {1 \over 2}(x^{(2)}-1)^2$ with $\nabla f(x) = (x^{(1)}, x^{(2)}-1)^T$. Define $\psi (x) = \textrm{Ind} \, Q$, the indicator function of $Q = \Big \{ x \in {\mathbb {R}}^2:\; x^{(2)} \le 0 \Big \}$. Then $f_*=0.5$. Consider the point $x_k= (x^{(1)}_k, 0)^T$. By the classical step-size rule, we have

$$\begin{aligned} \begin{array}{rcl} x_{k+1} &{} = &{} \pi _{Q} \left[ x_k - {f(x_k) - f_* \over \Vert \nabla f(x_k) \Vert ^2} \nabla f(x_k) \right] \; = \; \pi _{Q} \left[ \left( \begin{array}{c} x^{(1)}_k \\ 0 \end{array} \right) - {\left( x^{(1)}_k\right) ^2 \over 2\left( 1+ \left( x^{(1)}_k\right) ^2 \right) } \left( \begin{array}{c} x^{(1)}_k \\ - 1 \end{array} \right) \right] . \end{array} \end{aligned}$$

Thus, $x^{(2)}_{k+1} = 0$ and $x^{(1)}_{k+1} = x^{(1)}_k - {\left( x^{(1)}_k\right) ^3 \over 2\left( 1+ \left( x^{(1)}_k\right) ^2 \right) }$. This means that $x_k^{(1)} = O(k^{-1/2})$.

On the other hand, the rule (3.10) as applied to the same point $x_k$ defines $x = x_{k+1}$ as an intersection of two lines

$$\begin{aligned} \begin{array}{rcl} \Big \{x \in {\mathbb {R}}^2: \; \langle \nabla f(x_k), x_k - x \rangle = f(x_k) - f_* \Big \}, \quad \Big \{x\in {\mathbb {R}}^2: \; x^{(2)} = 0 \Big \}. \end{array} \end{aligned}$$

This means that $x^{(1)}_k \left( x^{(1)}_k - x^{(1)}_{k+1}\right) = \hbox { }\ {1 \over 2}\left( x^{(1)}_k\right) ^2$, and we get the linear rate of convergence to the optimal point $x_* = 0$. $\square$

## Step-size control for problems with smooth strongly convex components

A linear rate of convergence, demonstrated by method (3.7) in the Example 2, provides us with motivation to look at behavior of this method on smooth and strongly convex problems. Let us introduce the Euclidean metric by (1.5) and define $d(x) = \hbox { }\ {1 \over 2}\Vert x \Vert ^2_B$, $x \in {\mathbb {E}}$. In this case,

$$\begin{aligned} \begin{array}{rcl} \beta _d(x,y)= & {} \hbox { }\ {1 \over 2}\Vert x - y \Vert ^2_B, \quad x, y \in {\mathbb {E}}. \end{array} \end{aligned}$$

Suppose that all functions $f_i(\cdot )$, $i = 1, \dots , m$ in (3.2) are continuously differentiable and have Lipschitz-continuous gradients with the same constant $L_f \ge 0$:

4.1 $$\begin{aligned} \begin{array}{rcl} f_i(y)\le & {} f_i(x) + \langle \nabla f_i(x), y - x \rangle + \hbox { }\ {1 \over 2}L_f \Vert x - y \Vert ^2_B, \quad x, y \in \mathrm{dom \,}\psi . \end{array} \end{aligned}$$

In the notation of Sect. 3, these inequalities imply the following upper bound for the objective function of problem (3.1):

4.2 $$\begin{aligned} \begin{array}{rcl} F(y)\le & {} \ell _x(y) + \psi (y) + \hbox { }\ {1 \over 2}L_f \Vert x - y \Vert ^2_B, \quad x, y \in \mathrm{dom \,}\psi . \end{array} \end{aligned}$$

Hence, for the sequence of points $\{ x_k \}_{k \ge 0}$, generated by method (3.7), we have

4.3 $$\begin{aligned} \begin{array}{rcl} F(x_{k+1}) &{} \le &{} \ell _{x_k}(x_{k+1}) + \psi (x_{k+1}) + \hbox { }\ {1 \over 2}L_f \Vert x_k - x_{k+1} \Vert ^2_B \\ \\ &{} {\mathop {\le }\limits ^{(3.12)}} &{} F_* + \hbox { }\ {1 \over 2}L_f \Vert x_k - x_{k+1} \Vert ^2_B. \end{array} \end{aligned}$$

Thus, we can prove the following statement.

### Theorem 2

Under condition (4.1), the rate of convergence of method (3.7) can be estimated as follows:

4.4 $$\begin{aligned} \begin{array}{rcl} {1 \over T} \sum \limits _{k=1}^{T} F(x_k) - F_*\le & {} {1 \over 2T} L_f \Vert x_0 - x_* \Vert ^2_B, \quad T \ge 1. \end{array} \end{aligned}$$

If in addition functions $f_i(\cdot )$, $i = 1, \dots , m$, are strongly convex:

4.5 $$\begin{aligned} \begin{array}{rcl} f_i(y)\ge & {} f_i(x) + \langle \nabla f_i(x), y - x \rangle + \hbox { }\ {1 \over 2}\mu _f \Vert x - y \Vert ^2_B, \quad x, y \in \mathrm{dom \,}\psi , \end{array} \end{aligned}$$

with $\mu _f > 0$, then the rate of convergence is linear:

4.6 $$\begin{aligned} \begin{array}{rcl} \Vert x_k - x_* \Vert ^2_B\le & {} \left( {L_f \over \mu _f + L_f}\right) ^k \Vert x_0 - x_* \Vert ^2_B, \quad k \ge 0. \end{array} \end{aligned}$$

### Proof

Indeed, substituting inequality (4.3) into relation (3.11), we have

4.7 $$\begin{aligned} \begin{array}{rcl} \hbox { }\ {1 \over 2}\Vert x_{k+1} - x_* \Vert ^2_B\le & {} \hbox { }\ {1 \over 2}\Vert x_k - x_* \Vert ^2_B - {1 \over L_f} [ F(x_{k+1}) - F_*], \quad k \ge 0. \end{array} \end{aligned}$$

Summing up these inequalities for $k = 0, \dots , T-1$, we get inequality (4.4).

If in addition, the conditions (4.5) are satisfied, then

4.8 $$\begin{aligned} \begin{array}{rcl} F(y)\ge & {} \ell _x(y) + \psi (y) + \hbox { }\ {1 \over 2}\mu _f \Vert x - y \Vert ^2_B, \quad x, y \in \mathrm{dom \,}\psi . \end{array} \end{aligned}$$

Note that the first-order optimality conditions for problem (3.1) can be written in the following form:

$$\begin{aligned} \begin{array}{rcl} \ell _{x_*}(y) + \psi (y)\ge & {} F_*, \quad \forall y \in \mathrm{dom \,}\psi . \end{array} \end{aligned}$$

Therefore, inequality (4.8) implies that

$$\begin{aligned} \begin{array}{rcl} F(y) - F_*\ge & {} \hbox { }\ {1 \over 2}\mu _f \Vert y - x_* \Vert ^2_B, \quad \forall y \in \mathrm{dom \,}\psi . \end{array} \end{aligned}$$

Thus, $\Vert x_{k+1} - x_* \Vert ^2_B {\mathop {\le }\limits ^{(4.7)}} {L_f \over \mu _f + L_f} \Vert x_k - x_* \Vert ^2_B$, and we get inequality (4.6). $\square$

## Convex minimization with max-type composite constraint

Let us show that both step-size strategies described in Sects. 2 and 3 can be unified in one scheme for solving constrained optimization problems. In this section, we deal with the problem in the following *semi-composite* form:

5.1 $$\begin{aligned} \begin{array}{rcl} \min \limits _{x \in Q} \Big \{ f_0(x): \; F(x) {\mathop {=}\limits ^{\textrm{def}}}f(x) + \psi (x) \le 0 \Big \} \end{array} \end{aligned}$$

where $\psi (\cdot )$ is a simple closed convex function, $Q \subseteq \mathrm{dom \,}\psi$ is a closed convex set, and

$$\begin{aligned} \begin{array}{rcl} f(x)= & {} \max \limits _{1 \le i \le m} f_i(x), \end{array} \end{aligned}$$

with all functions $f_i(\cdot )$, $i = 0, \dots , m$, being closed and convex on $\mathrm{dom \,}\psi$. We assume *Q* to be bounded:

5.2 $$\begin{aligned} \begin{array}{rcl} \beta _d(x,y)< & {} D, \quad \forall x, y \in Q. \end{array} \end{aligned}$$

In order to solve problem (5.1), we propose a method, which combines two different types of iterations. One of them improves the *feasibility* of the current point, and the second one improves its *optimality*.

Iteration of the first type is based on the machinery developed in Sect. 3 with the particular value $F_* = 0$. It is applied to some point $x_k \in Q$.5.3Note that for $F(x_k) \le 0$, we have $\lambda _k = 0$ and ${{\hat{T}}}_{x_k}(\lambda _k) = x_k$.

For iteration *k* of the second type, we need to choose a primal step-size bound $h_k > 0$. Then, at the test point $y_k \in Q$, we define the function $\varphi _{y_k}(\cdot )$ by (2.7) and apply the following rule:5.4Since in both rules parameters $\lambda _k$ are functions of the test points, we will use shorter notations $T(y_k)$ and ${{\hat{T}}}(x_k)$. Consider the following optimization scheme.5.5Thus, method (5.5) is defined by a sequence of primal step bounds ${{{\mathcal {H}}}} = \{ h_k \}_{k \ge 0}$. However, since in (5.5) we apply a switching strategy, it is impossible to say in advance what will be the type of a particular *k*th iteration. Therefore, as compared with the classical conditions (2.12), we need additional regularity assumptions on ${{{\mathcal {H}}}}$.

It will be convenient to relate this sequence with another sequence of *scaling coefficients* ${{{\mathcal {T}}}} = \{ \tau _k \}_{k\ge 0}$, satisfying the *second-order divergence condition* (compare with (2.12)).5.6Note that condition (5.6) ensures $\sum \limits _{k=0}^{\infty } \tau _k = + \infty$. Thus, it is stronger than (2.12). In order to transform the sequence $\mathcal{T}$ into *convergence rate* of some optimization process, we need to introduce the following characteristic.

### Definition 1

For a sequence ${{{\mathcal {T}}}}$, the integer-valued function *a*(*k*), $k \ge 0$, is called the *divergence delay* (of degree two) if $a(k) \ge 0$ is the minimal integer value such that

5.7 $$\begin{aligned} \begin{array}{rcl} \sum \limits _{i=k}^{k+a(k)} \tau _i^2\ge & {} 1, \quad k \ge 0. \end{array} \end{aligned}$$

Clearly, condition (5.6)_b_ ensures that all values *a*(*k*), $k \ge 0$, are well defined. At the same time, from (5.6)_a_, we have $a(k+1) \ge a(k)$ for any $k \ge 0$.

Let us give two important examples of such sequences.

### Example 3

**(a)** Let us fix an integer $N \ge 0$ and define

5.8 $$\begin{aligned} \begin{array}{rcl} \tau _k= & {} {1 \over \sqrt{N+1}}, \quad k \ge 0. \end{array} \end{aligned}$$

Then condition (5.6)_a_ is valid and $\sum \limits _{i=k}^{k+a(k)} \tau _i^2 = {a(k)+1 \over N+1}$. Thus, $a(k)= N$.

**(b)** Consider the following sequence:

5.9 $$\begin{aligned} \begin{array}{rcl} \tau _k= & {} \sqrt{2 \over k+1}, \quad k \ge 0. \end{array} \end{aligned}$$

For $k \ge 1$, denote by $S_k = \sum \limits _{i=0}^{k-1} \tau _i^2$. Then $S_1 = 2$, $S_2 = 3$, and the difference

$$\begin{aligned} \begin{array}{rcl} S_{2k} - S_k= & {} \sum \limits _{i=k}^{2k-1} \tau _i^2 \end{array} \end{aligned}$$

is monotonically increasing in $k \ge 1$. Thus, $a(k) \le k-1$ for all $k \ge 1$. $\square$

Let us analyze the performance of the method (5.5) with an appropriately chosen sequence ${{{\mathcal {H}}}}$. Namely, let us choose

5.10 $$\begin{aligned} \begin{array}{rcl} h_k= & {} \sqrt{2D} \; \tau _k, \quad k \ge 0, \end{array} \end{aligned}$$

where the sequence ${{{\mathcal {T}}}}$ satisfies condition (5.6) and *D* is taken from (5.2).

We are interested only in the values of objective function $f_0(\cdot )$ computed at the points with small values of the functional constraint $F(\cdot )$. As we will see, these points are involved in Step 2b). For the total number of steps $N \ge 1 + a(0)$, denote

5.11 $$\begin{aligned} \begin{array}{rcl} k(N) &{} = &{} \max \Big \{ k \ge 0: \; k + a(k) \le N-1\Big \},\\ \\ {{{\mathcal {F}}}}_N &{} = &{} \Big \{ k: \; k(N) \le k \le N-1, \; y_k {\mathop {\longrightarrow }\limits ^{2b)}} x_{k+1} \Big \}. \end{array} \end{aligned}$$

Let us define the following directional proximity measures:

$$\begin{aligned} \begin{array}{rclll} \delta _k= & {} \delta _{s_k}(y_k)&\hbox {with }\; s_k = {y_k - x_{k+1} \over \Vert y_k - x_{k+1} \Vert },&k \in {{{\mathcal {F}}}}_N. \end{array} \end{aligned}$$

We are interested in the rate of convergence to zero of the following characteristic:

$$\begin{aligned} \begin{array}{rcl} \delta _N^*= & {} \min \limits _{k \in {{{\mathcal {F}}}}_N} \delta _k. \end{array} \end{aligned}$$

### Theorem 3

For any $N \ge 1 + a(0)$, the number $k(N)\ge 0$ is well defined and $\delta _N^* < h_{k(N)}$.

Moreover, if all functions $f_i(\cdot )$, $i = 1, \dots , m$, have Hölder-continuous gradients on *Q* with parameter $\nu \in [0,1]$ and constant $L_{\nu }>0$, then, for any $k \in \mathcal{F}_N$, we have

5.12 $$\begin{aligned} \begin{array}{rcl} F(y_k)\le & {} {L_{\nu } \over 1 + \nu } h_{k(N)}^{1+\nu }. \end{array} \end{aligned}$$

### Proof

Let us bound the distances $r_k = \beta _d(x_k,x_*)$ for $k \ge k(N)$. If $k \not \in {{{\mathcal {F}}}}_N$, then

$$\begin{aligned} \begin{array}{rcl} r_{k+1}= & {} \beta _d(y_k,x_* ) \; {\mathop {\le }\limits ^{(3.11)}} \; r_k - \beta (x_k,y_k ) \; {\mathop {\le }\limits ^{(5.5)}} \; r_k - \hbox { }\ {1 \over 2}h_k^2. \end{array} \end{aligned}$$

If $$k \in {{{\mathcal {F}}}}_N$$, then

$$\begin{aligned} \begin{array}{rcl} r_{k+1}&{\mathop {\le }\limits ^{(2.17)}}&\beta (y_k,x_*) - h_k \delta _k + \hbox { }\ {1 \over 2}h_k^2 \; {\mathop {\le }\limits ^{(3.11)}} \; r_k - h_k \delta _k + \hbox { }\ {1 \over 2}h_k^2 \; \le \;r_k - h_k \delta ^*_N + \hbox { }\ {1 \over 2}h_k^2. \end{array} \end{aligned}$$

Summing up these inequalities for $k = k(N), \dots N-1$, we obtain

$$\begin{aligned} r_{N}{} & {} < D - \hbox { }\ {1 \over 2}\sum \limits _{k \not \in {{{\mathcal {F}}}}_N} h_k^2 + \hbox { }\ {1 \over 2}\sum \limits _{k \in {{{\mathcal {F}}}}_N} ( h_k^2 - 2 h_k \delta ^*_N)\\ \\{} & {} = D - \hbox { }\ {1 \over 2}\sum \limits _{k=k(N)}^{N-1} h_k^2 + \sum \limits _{k \in {{{\mathcal {F}}}}_N} ( h_k^2 - h_k \delta ^*_N)\\ \\{} & {} {\mathop {<}\limits ^{(5.7),(5.10)}} \sum \limits _{k \in {{{\mathcal {F}}}}_N} h_k ( h_k - \delta ^*_N) \; {\mathop {\le }\limits ^{(5.6)_a}} \; ( h_{k(N)} - \delta ^*_N) \sum \limits _{k \in {{{\mathcal {F}}}}_N} h_k. \end{aligned}$$

Thus, $\delta ^*_N < h_{k(N)}$. Finally, inclusion $k \in {{{\mathcal {F}}}}_N$ implies

$$\begin{aligned} \begin{array}{rcl} \hbox { }\ {1 \over 2}\Vert y_k - x_k \Vert ^2&{\mathop {\le }\limits ^{(1.8)}}&\beta _d(x_k,y_k) \; \le \; \hbox { }\ {1 \over 2}h_k^2 \; {\mathop {\le }\limits ^{(5.6)_a}} \; \hbox { }\ {1 \over 2}h_{k(N)}^2. \end{array} \end{aligned}$$

Since $F_* = 0$, we get

$$\begin{aligned} F(y_k){} & {} \le \ell _{x_k}(y_k) + {L_\nu \over 1+\nu } \Vert y_k - x_k \Vert ^{1+\nu } + \psi (y_k)\\ \\{} & {} {\mathop {\le }\limits ^{(3.12)}} {L_\nu \over 1+\nu } \Vert y_k - x_k \Vert ^{1+\nu } \; \le \; {L_\nu \over 1+\nu } h_{k(N)}^{1+\nu }, \end{aligned}$$

and this is inequality (5.12). $\square$

As a straightforward consequence of Theorem 3, we have the following rate of convergence in function value:

5.13 $$\begin{aligned} \begin{array}{rcl} \min \limits _{x \in {{{\mathcal {F}}}}_N} f_0(y_k)\le & {} f^*_0 + \mu (h_{k(N)}), \end{array} \end{aligned}$$

where the function $\mu (\cdot )$ is defined by (2.4).

Thus, the actual rate of convergence of the method (5.5) depends on the rate of convergence of sequence ${{{\mathcal {T}}}}$ and the magnitude of divergence delay. For example, for the choice (5.9), in view of inequality $a(k) \le k-1$, we have

$$\begin{aligned} \begin{array}{rcl} k(N)= & {} \max \Big \{ k: \; k + a(k) \le N-1\Big \} \; \ge \; \max \Big \{ k: \;2k-1 \le N-1\Big \}. \end{array} \end{aligned}$$

Hence, for $N = 2M$, the choice (5.9) ensures $k(N) \ge M = N/2$, and we have

5.14 $$\begin{aligned} \begin{array}{rcl} h_{k(N)}\le & {} h_M \; = \; {2 D^{1/2} \over \sqrt{M + 1}}. \end{array} \end{aligned}$$

It is interesting that the rate of convergence (5.12) for the constraints can be higher than the rate (5.13) for the objective function.

## Approximating Lagrange Multipliers, I

Despite the good convergence rate (5.13), when the number of functional components *m* in problem (5.1) is big, the implementation of one iteration (5.3) can be very expensive. In this section, we consider a simpler switching strategy for solving convex optimization problems with potentially many functional constraints. Our method is also able to approximate the corresponding optimal Lagrange multipliers.

Consider the following *constrained* optimization problem:

6.1 $$\begin{aligned} \begin{array}{rcl} f_0^* \; = \; \min \limits _{x \in Q} \Big \{ f_0(x): \; f_i(x) \le 0, \; i = 1, \dots , m \Big \}, \end{array} \end{aligned}$$

where all functions $f_i(\cdot )$ are closed and convex, $i = 0, \dots , m$, and set *Q* is closed, convex, and bounded. Denote by $x^*$ one of its optimal solutions. Sometimes we use notation $\bar{f}(x) = (f_1(x), \dots , f_m(x))^T \in {\mathbb {R}}^m$. Denote ${{{\mathcal {F}}}} = \{ x \in Q: \; {{\bar{f}}}(x) \le 0 \}$.

We assume that functions $f_i(\cdot )$ are subdifferentiable on *Q* and it is possible to compute their subgradients with uniformly bounded norms:

6.2 $$\begin{aligned} \begin{array}{rcl} \Vert f'_i(x) \Vert _*\le & {} M_i, \quad x \in Q, \quad i = 0, \dots , m. \end{array} \end{aligned}$$

Denote $${{\bar{M}}} = (M_1, \dots , M_m)^T \in {\mathbb {R}}^m$$.

For the set *Q*, we assume existence of a prox-function $d(\cdot )$ defining the corresponding Bregman distance $\beta _d(\cdot ,\cdot )$. In our methods, we need to know a constant *D* such that

6.3 $$\begin{aligned} \begin{array}{rcl} \beta _d(x,y)< & {} D, \quad \forall x, y \in Q. \end{array} \end{aligned}$$

Let us introduce the Lagrangian

$$\begin{aligned} \begin{array}{rcl} {{{\mathcal {L}}}}(x,{{\bar{\lambda }}})= & {} f_0(x) + \langle {{\bar{\lambda }}}, {{\bar{f}}}(x) \rangle , \quad {{\bar{\lambda }}} = (\lambda ^{(1)}, \dots , \lambda ^{(m)})^T \in {\mathbb {R}}^m_+, \end{array} \end{aligned}$$

and the dual function $\phi ({{\bar{\lambda }}}) = \min \limits _{x \in Q} {{{\mathcal {L}}}}(x,{{\bar{\lambda }}})$. By Sion’s theorem, we know that

6.4 $$\begin{aligned} \begin{array}{rcl} \sup \limits _{{{\bar{\lambda }}} \in {\mathbb {R}}^m_+} \phi ({{\bar{\lambda }}})= & {} f_0^*. \end{array} \end{aligned}$$

Our first method is based on two operations presented in Sects. 2 and 3. For defining iterations of the first type, we need functions $\varphi _{i,x}(\lambda )$ with $\lambda \ge 0$, parameterized by $x \in Q$, and defined as follows:

6.5 $$\begin{aligned} \begin{array}{rcl} \varphi _{i,x}(\lambda )= & {} \max \limits _{T \in Q} \Big \{ \lambda \langle f'_i(x), x - T \rangle - \beta _d(x,T) \Big \}, \quad i = 0, \dots , m. \end{array} \end{aligned}$$

An appropriate value of $\lambda$ can be found from the equation

6.6 $$\begin{aligned} \begin{array}{rcl} \varphi _{i,x_k}(\lambda )= & {} \hbox { }\ {1 \over 2}h_k^2, \end{array} \end{aligned}$$

and used for setting $x_{k+1} = T_{i,x_k}(\lambda )$, the optimal solutions of problem (6.5). In this section, we perform this iteration only for the objective function ($i=0$). The possibility of using the steps $T_{i,x_k}(\lambda )$ for inequality constraints is analyzed in Sects. 7 and 9.

The iteration of the second type is trying to improve feasibility of the current point $x_k \in Q$ (compare with (5.3)). It needs computation of all Bregman projections

6.7 $$\begin{aligned} \begin{array}{rcl} {{\hat{T}}}_i(x_k)&{\mathop {=}\limits ^{\textrm{def}}}&\arg \min \limits _{T \in Q} \Big \{ \beta _d(x_k,T): \; f_i(x_k) + \langle f'_i(x_k), T - x_k \rangle \le 0 \Big \}, \end{array} \end{aligned}$$

for indexes $i = 1, \dots , m$. The first-order optimality condition for problem (6.7) is as follows:

6.8 $$\begin{aligned} \begin{array}{rcl} \langle \nabla d(T) - \nabla d(x_k) + \lambda f'_i(x_k), x - T \rangle\ge & {} 0, \quad \forall x \in Q, \end{array} \end{aligned}$$

where $T = {{\hat{T}}}_i(x_k)$ and $\lambda = \lambda _i(x_k) \ge 0$ is the optimal Lagrange multiplier for the linear inequality constraint in (6.7). We assume that this multiplier can be also computed. Note that for $x = x_k$ we have

$$\begin{aligned} \begin{array}{rcl} \lambda _i(x_k) \langle f'_i(x_k), x_k - T \rangle\ge & {} \beta _d(x_k,T) + \beta _d(T,x_k) \; {\mathop {\ge }\limits ^{(1.8)}} \; \Vert x_k - T \Vert ^2. \end{array} \end{aligned}$$

Hence, if $T \ne x_k$, then $\lambda _i(x_k) > 0$.

Consider the following optimization scheme.6.9Let us prove that method (6.9) can find an approximate solution of the primal-dual problem (6.1), (6.4). Let us choose a sequence ${{{\mathcal {T}}}}$ satisfying condition (5.6) and define

6.10 $$\begin{aligned} \begin{array}{rcl} h_k= & {} \sqrt{2D} \; \tau _k, \quad k \ge 0, \end{array} \end{aligned}$$

where *D* satisfies inequality (6.3). Then, for the number of steps $N \ge 1 + a(0)$, let us define function *k*(*N*) by the first equation in (5.11). Now we can define the following objects:

6.11 $$\begin{aligned} \begin{array}{rcl} {{{\mathcal {A}}}}_i(N) &{} = &{} \{k: \; k(N) \le k \le N-1, \; i_k = i \}, \\ \\ \sigma _i(N) &{} = &{} \sum \limits _{k \in {{{\mathcal {A}}}}_i(N)} \lambda _k, \quad i = 0, \dots , m,\\ \\ \lambda ^*_i(N) &{} = &{} \sigma _i(N)/\sigma _0(N), \quad i = 1, \dots , m. \end{array} \end{aligned}$$

Clearly, the vectors of dual multipliers ${{\bar{\lambda }}}_*(N) = (\lambda ^*_1(N), \dots , \lambda ^*_m(N))$ are defined only if $\mathcal{A}_0(N) \ne \emptyset$. As usual, the sum over an empty set of iterations is assumed to be zero.

### Theorem 4

Let the sequence of step bounds ${{{\mathcal {H}}}}$ in method (6.9) be defined by (6.10). Then for any $N \ge 1 + a(0)$, we have ${{{\mathcal {A}}}}_0(N) \ne \emptyset$. Moreover, if all functions $f_i(\cdot )$, $0 = 1, \dots , m$, satisfy (6.2), then

6.12 $$\begin{aligned} \begin{array}{rcl} \max \limits _{1 \le i \le m} {1 \over M_i} f_i(x_k)\le & {} h_{k(N)}, \quad k \in {{{\mathcal {A}}}}_0(N), \end{array} \end{aligned}$$

6.13 $$\begin{aligned} \begin{array}{rcl} {1 \over \sigma _0(N)} \sum \limits _{k \in {{{\mathcal {A}}}}_0(N)} \lambda _k f_0(x_k)\le & {} \phi ({{\bar{\lambda }}}^*(N)) + M_0 h_{k(N)}. \end{array} \end{aligned}$$

### Proof

Indeed, we have

$$\begin{aligned} \begin{array}{rcl} B_N &{} {\mathop {=}\limits ^{\textrm{def}}}&{} \max \limits _{x \in Q} \Big [ \sum \limits _{k \in {{{\mathcal {A}}}}_0(N)} \lambda _k f_0(x_k) - \sigma _0(N) f_0(x) - \sum \limits _{i=1}^m \sigma _i(N) f_i(x) \Big ]\\ \\ &{} = &{} \max \limits _{x \in Q} \Big [ \sum \limits _{k \in {{{\mathcal {A}}}}_0(N)} \lambda _k [f_0(x_k) - f_0(x)] - \sum \limits _{i=1}^m \sigma _i(N) f_i(x) \Big ]. \end{array} \end{aligned}$$

Let us fix some $x \in Q$ and denote $r_k(x) = \beta _d(x_k,x)$. Then, for $k \in {{{\mathcal {A}}}}_0(N)$, we have

6.14 $$\begin{aligned} \begin{array}{rcl} \lambda _k [f_0(x_k) - f_0(x)]\le & {} \lambda _k \langle f'_0(x_k), x_k - x \rangle . \end{array} \end{aligned}$$

At the same time, from the first-order optimality condition for problem (6.5), we have

$$\begin{aligned} \begin{array}{rl} 0 &{}\le \langle \nabla d(x_{k+1}) - \nabla d(x_k), x - x_{k+1} \rangle + \lambda _k \langle f'_0(x_k), x - x_{k+1} \rangle \\ \\ &{} {\mathop {\le }\limits ^{(6.14)}} \langle \nabla d(x_{k+1}) - \nabla d(x_k), x - x_{k+1} \rangle - \lambda _k [f_0(x_k) - f_0(x)] + \lambda _k \langle f'_0(x_k), x_k - x_{k+1} \rangle \\ \\ &{}= \langle \nabla d(x_{k+1}) - \nabla d(x_k), x - x_{k+1} \rangle - \lambda _k [f_0(x_k) - f_0(x)] + \varphi _{0,x_k}(\lambda _k) + \beta _d(x_k,x_{k+1}). \end{array} \end{aligned}$$

Hence,

$$\begin{aligned} \begin{array}{rl} &{} r_{k+1}(x) - r_k(x) \; = \; \langle \nabla d(x_k) - \nabla d(x_{k+1}), x - x_{k+1} \rangle - \beta _d(x_k,x_{k+1})\\ \\ &{}\quad \le - \lambda _k [f_0(x_k) - f_0(x)] + \varphi _{0,x_k}(\lambda _k) \; {\mathop {=}\limits ^{(6.6)}} \; - \lambda _k [f_0(x_k) - f_0(x)] + \hbox { }\ {1 \over 2}h_k^2. \end{array} \end{aligned}$$

On the other hand,

$$\begin{aligned} \sum \limits _{i=1}^m \sigma _i(N) f_i(x){} & {} {\mathop {=}\limits ^{(6.11)}} \sum \limits _{i=1}^m \sum \limits _{k \in {{{\mathcal {A}}}}_i(N)} \lambda _k f_i(x) \; = \; \sum \limits _{k \not \in {{{\mathcal {A}}}}_0(N)} \lambda _{i_k}(x_k) f_{i_k}(x)\\ \\{} & {} \ge \sum \limits _{k \not \in {{{\mathcal {A}}}}_0(N)} \lambda _{i_k}(x_k) [f_{i_k}(x_k) + \langle f'_{i_k}(x_k), x - x_k \rangle ]\\ \\{} & {} {\mathop {=}\limits ^{(6.7)}} \sum \limits _{k \not \in {{{\mathcal {A}}}}_0(N)} \lambda _{i_k}(x_k) [\langle f'_{i_k}(x_k), x - x_{k+1} \rangle ] \\ \\{} & {} {\mathop {\ge }\limits ^{(6.8)}} \sum \limits _{k \not \in {{{\mathcal {A}}}}_0(N)} \langle \nabla d(x_{k+1}) - \nabla d(x_k), x_{k+1} - x \rangle \\ \\{} & {} = \sum \limits _{k \not \in {{{\mathcal {A}}}}_0(N)} \Big [ r_{k+1}(x) - r_{k}(x) + \beta _d(x_k,x_{k+1}) \Big ] \\ \\{} & {} {\mathop {\ge }\limits ^{(6.9)_2}} \sum \limits _{k \not \in \mathcal{A}_0(N)} \Big [ r_{k+1}(x) - r_{k}(x) + \hbox { }\ {1 \over 2}h_k^2 \Big ]. \end{aligned}$$

Therefore,

$$\begin{aligned} \begin{array}{rcl} B_N &{} \le &{} \max \limits _{x \in Q} \Big [ \sum \limits _{k \in {{{\mathcal {A}}}}_0(N)} [r_k(x) - r_{k+1}(x) + \hbox { }\ {1 \over 2}h_k^2] - \sum \limits _{k \not \in {{{\mathcal {A}}}}_0(N)} [ r_{k+1}(x) - r_{k}(x) + \hbox { }\ {1 \over 2}h_k^2 ] \; \Big ]\\ \\ &{} = &{} \max \limits _{x \in Q} \Big [ r_{k(N)}(x) - r_N(x) + \hbox { }\ {1 \over 2}\sum \limits _{k \in {{{\mathcal {A}}}}_0(N)} h_k^2 - \hbox { }\ {1 \over 2}\sum \limits _{k \not \in {{{\mathcal {A}}}}_0(N)} h_k^2 \Big ] \\ \\ &{} < &{} D - \hbox { }\ {1 \over 2}\sum \limits _{k=k(N)}^{N-1} h_k^2 + \sum \limits _{k \in {{{\mathcal {A}}}}_0(N)} h_k^2 \; {\mathop {\le }\limits ^{(6.10)}} \; \sum \limits _{k \in {{{\mathcal {A}}}}_0(N)} h_k^2 \; {\mathop {\le }\limits ^{(5.6)_a}} \; h_{k(N)} \sum \limits _{k \in {{{\mathcal {A}}}}_0(N)} h_k. \end{array} \end{aligned}$$

Let us assume now that ${{{\mathcal {A}}}}_0(N) = \emptyset$. Then $B_N < 0$, and this is impossible since the point $x_*$ is feasible. Thus, we have proved that ${{{\mathcal {A}}}}_0(N) \ne \emptyset$.

Further, for any $k \in {{{\mathcal {A}}}}_0(N)$ we have

$$\begin{aligned} \begin{array}{rcl} \lambda _k \langle f'_0(x_k), x_k - x_{k+1} \rangle&{\mathop {=}\limits ^{(6.6)}}&\beta _d(x_k,x_{k+1}) + \hbox { }\ {1 \over 2}h_k^2. \end{array} \end{aligned}$$

Hence, $r_k^0 = \Vert x_{k+1} - x_k \Vert > 0$ and we conclude that

$$\begin{aligned} \begin{array}{rcl} \lambda _k M_0&{\mathop {\ge }\limits ^{(6.2)}} \lambda _k \Vert f'_0(x_k) \Vert _* \; \ge {\lambda _k \over r_k^0} \langle f'_0(x_k), x_k - x_{k+1} \rangle \; {\mathop {\ge }\limits ^{(1.8)}} \; {1 \over 2 r_k^0} \Big [ (r_k^0)^2 + h_k^2 \Big ] \; \ge \; h_k. \end{array} \end{aligned}$$

Thus, we have proved that $B_N \le M_0 \sigma _0(N) h_{k(N)}$. Dividing this inequality by $\sigma _0(N) > 0$, we get the bound (6.13).

Finally, note that for any $k \in {{{\mathcal {A}}}}_0(N)$ we have $\Vert {{\hat{T}}}_i(x_k) - x_k \Vert \le h_k$ for all *i*, $1 \le i \le m$. Note that either $f_i(x_k) \le 0$, or $f_i(x_k) > 0$ and $f_i(x_k) + \langle f'_i(x_k), {{\hat{T}}}_i(x_k) - x_k) \rangle = 0$. In the latter case, we have

$$\begin{aligned} \begin{array}{rcl} f_i(x_k)= & {} \langle f'_i(x_k), x_k - {{\hat{T}}}_i(x_k)\rangle \; {\mathop {\le }\limits ^{(6.2)}} \; M_i h_k \; \le \; M_i h_{k(N)}. \end{array} \end{aligned}$$

Thus, inequality (6.12) is proved. $\square$

Note that for the choice of scaling sequence (5.9), method (5.5) is globally convergent. In this case, the computation of Lagrange multipliers by (6.11) for all values of *N*, requires storage of all coefficients $\{ \lambda _k \}_{k \ge 0}$. This inconvenience can be avoided if we decide to accumulate the sums for Lagrange multipliers only starting from the moments $k(N_q)$ with $N_q = 2^q$, $q \ge 1$. Then the method (5.5) will be allowed to stop only at the moments $2N_q$.

## Approximating Lagrange Multipliers, II

Method (6.9) has one hidden drawback. If $i_k \ge 1$, then

$$\begin{aligned} \begin{array}{rcl} 0&{\mathop {=}\limits ^{(6.7)}}&f_{i_k}(x_k) + \langle f'_{i_k}(x_k), x_{k+1} - x_k \rangle \; \le \; f_{i_k}(x_{k+1}). \end{array} \end{aligned}$$

Thus, this scheme most probably generates infeasible approximations of the optimal point, which violate some of the functional constraints. In order to avoid this tendency, we propose a scheme which uses for both types of iterations (improving either feasibility or optimality) the same step-size rule (6.6).7.1Thus, for both types of iterations, we use the same step-size strategy (6.6). Note that for any $i = 0, \dots , m$ and $T_i = T_{i,x_k}(\lambda _{i,k})$, we have

$$\begin{aligned} \begin{array}{rcl} \lambda _{i,k} \langle f'_i(x_k), x_k - T_i \rangle &{} {\mathop {=}\limits ^{(6.5)}} &{} \varphi _{i,x_k}(\lambda _{i,k}) + \beta _d(x_k,T_i) \; {\mathop {=}\limits ^{(6.6)}} \; \hbox { }\ {1 \over 2}h_k^2 + \beta _d(x_k,T_i)\\ \\ &{} {\mathop {\ge }\limits ^{(1.8)}} &{} \hbox { }\ {1 \over 2}h_k^2 + \hbox { }\ {1 \over 2}\Vert x_k - T_i \Vert ^2 \; \ge \; h_k \Vert x_k - T_i \Vert . \end{array} \end{aligned}$$

Hence, since $T_i \ne x_k$, we have

7.2 $$\begin{aligned} \begin{array}{rcl} \lambda _{i,k} \Vert f'_{i}(x_k) \Vert _*\ge & {} h_k, \quad k \ge 0. \end{array} \end{aligned}$$

In method (7.1), we choose ${{{\mathcal {H}}}}$ in accordance to (6.10). Then, for $N \ge 1 + a(0)$, we define function *k*(*N*) by the first equation in (5.11) and introduce by (6.11) the approximations of the optimal Lagrange multipliers.

### Theorem 5

Let the sequence of points $\{ x_k \}_{k \ge 0}$ be generated by method (7.1). Then for any $N \ge 1 + a(0)$ the set ${{{\mathcal {A}}}}_0(N)$ is not empty. Moreover, if all functions $f_i(\cdot )$, $i = 1, \dots , m$, satisfy (6.2), then for all $k \in {{{\mathcal {A}}}}_0(N)$ we have

7.3 $$\begin{aligned} \begin{array}{rcl} f_i(x_k)\le & {} \Vert f'_{i}(x_k) \Vert _* h_k \; {\mathop {\le }\limits ^{(6.2)}} \; M_i h_{k(N)}, \quad i = 1, \dots , m, \end{array} \end{aligned}$$

7.4 $$\begin{aligned} \begin{array}{rcl} {1 \over \sigma _0(N)} \sum \limits _{k \in {{{\mathcal {A}}}}_0(N)} \lambda _k f_0(x_k)\le & {} \phi ({{\bar{\lambda }}}^*(N)) + M_0 h_{k(N)}. \end{array} \end{aligned}$$

### Proof

As in the proof of Theorem 4, for $x \in Q$, we denote $r_k(x) = \beta _d(x_k,x)$, and

$$\begin{aligned} \begin{array}{rcl} B_N &{} {\mathop {=}\limits ^{\textrm{def}}}&{} \max \limits _{x \in Q} \Big [ \sum \limits _{k \in {{{\mathcal {A}}}}_0(N)} \lambda _k f_0(x_k) - \sigma _0(N) f_0(x) - \sum \limits _{i=1}^m \sigma _i(N) f_i(x) \Big ]\\ \\ &{} = &{} \max \limits _{x \in Q} \Big [ \sum \limits _{k \in {{{\mathcal {A}}}}_0(N)} \lambda _k [f_0(x_k) - f_0(x)] - \sum \limits _{i=1}^m \sigma _i(N) f_i(x) \Big ]. \end{array} \end{aligned}$$

Then, for $k \in {{{\mathcal {A}}}}_0(N)$, we have proved that

$$\begin{aligned} \begin{array}{rcl} r_{k+1}(x) - r_k(x)\le & {} - \lambda _k [f_0(x_k) - f_0(x)] + \hbox { }\ {1 \over 2}h_k^2. \end{array} \end{aligned}$$

On the other hand,

$$\begin{aligned} \begin{array}{rcl} \sum \limits _{i=1}^m \sigma _i(N) f_i(x) &{} {\mathop {=}\limits ^{(6.11)}} &{} \sum \limits _{i=1}^m \sum \limits _{k \in {{{\mathcal {A}}}}_i(N)} \lambda _k f_i(x) \; = \; \sum \limits _{k \not \in {{{\mathcal {A}}}}_0(N)} \lambda _{i_k,k} f_{i_k}(x)\\ \\ &{} \ge &{} \sum \limits _{k \not \in {{{\mathcal {A}}}}_0(N)} \lambda _{i_k,k} [f_{i_k}(x_k) + \langle f'_{i_k}(x_k), x - x_k \rangle ]\\ \\ &{} {\mathop {\ge }\limits ^{(7.1)_2}} &{} \sum \limits _{k \not \in \mathcal{A}_0(N)} \Big [ h_k^2 + \lambda _{i_k,k} \langle f'_{i_k}(x_k), x - x_k \rangle \Big ]. \end{array} \end{aligned}$$

Note that the first-order optimality condition for problem (2.7) is as follows:

$$\begin{aligned} \begin{array}{rcl} \langle \nabla d(T_i) - \nabla d(x_k) + \lambda f'_i(x_k), x - T_i \rangle\ge & {} 0, \quad \forall x \in Q, \end{array} \end{aligned}$$

where $T_i$ is its optimal solution, Therefore, for all $x \in Q$ an $k \not \in {{{\mathcal {A}}}}_0(N)$, we have

$$\begin{aligned} \begin{array}{rl} \lambda _{i_k,k} \langle f'_{i_k}(x_k), x - x_k \rangle \; &{}= \; \lambda _{i_k,k} \Big [ \langle f'_{i_k}(x_k), x - x_{k+1} \rangle + \langle f'_{i_k}(x_k), x_{k+1} - x_k \rangle \Big ]\\ \\ &{} \ge \lambda _{i_k,k} \langle f'_{i_k}(x_k), x_{k+1} - x_k \rangle + \langle \nabla d(x_{k+1}) - \nabla d(x_k), x_{k+1} - x \rangle \\ \\ &{} = \lambda _{i_k,k} \langle f'_{i_k}(x_k), x_{k+1} - x_k \rangle + r_{k+1}(x) - r_{k}(x) + \beta _d(x_k,x_{k+1})\\ \\ &{} = r_{k+1}(x) - r_{k}(x) - \varphi _{i_k,x_k}(\lambda _{i,k}) \; {\mathop {=}\limits ^{(6.6)}} \; r_{k+1}(x) - r_{k}(x) - \hbox { }\ {1 \over 2}h_k^2. \end{array} \end{aligned}$$

MThus, as in the proof of Theorem 4, we conclude that

$$\begin{aligned} \begin{array}{rcl} B_N &{} \le &{} \max \limits _{x \in Q} \Big [ \sum \limits _{k \in {{{\mathcal {A}}}}_0(N)} [r_k(x) - r_{k+1}(x) + \hbox { }\ {1 \over 2}h_k^2] - \sum \limits _{k \not \in {{{\mathcal {A}}}}_0(N)} [ r_{k+1}(x) - r_{k}(x) + \hbox { }\ {1 \over 2}h_k^2 ] \; \Big ]\\ \\ &{} \le &{} h_{k(N)} \sum \limits _{k \in {{{\mathcal {A}}}}_0(N)} h_k \; {\mathop {\le }\limits ^{(7.2)}} \; M_0 h_{k(N)} \sigma _0(N). \end{array} \end{aligned}$$

Since ${{{\mathcal {A}}}}_0(N) \ne \emptyset$ (see the proof of Theorem 4), dividing this inequality by $\sigma _0(N) > 0$, we get inequality (7.4).

Finally, note that for all $k \in {{{\mathcal {A}}}}_0(N)$ and $i = 1, \dots , m$, we have

$$\begin{aligned} \begin{array}{rcl} \lambda _{i,k} f_i(x_k)\le & {} h_k^2 \; {\mathop {\le }\limits ^{(7.2)}} \; \lambda _{i,k} \Vert f'_i(x_k) \Vert _* h_k \; {\mathop {\le }\limits ^{(6.2)}} \; \lambda _{i,k} M_i h_k \; \le \; \lambda _{i,k} M_i h_{k(N)}. \end{array} \end{aligned}$$

Thus, inequality (7.3) is proved. $\square$

## Accuracy guarantees for the dual problem

In Sects. 6 and 7, we developed two convergent methods (6.9) and (7.1), which are able to approach the optimal solution of the primal problem (6.1), generating in parallel an approximate solution of the dual problem (6.4). Indeed, for $N \ge 1 + a(0)$, denote

$$\begin{aligned} \begin{array}{rcl} f_0^*(N)= & {} \min \limits _{k \in {{{\mathcal {A}}}}_0(N)} f_0(x_k), \quad x^*_N \; = \; \arg \min \limits _{x} \Big \{ f_0(x): \; x = x_k, \; k \in {{{\mathcal {A}}}}_0(N) \Big \}. \end{array} \end{aligned}$$

Then, in view of Theorems 4 and 5, we have

8.1 $$\begin{aligned} \begin{array}{rcl} f_0^*(N) - \phi ({{\bar{\lambda }}}^*(N)) &{} \le &{} M_0 h_{k(N)},\\ \\ \max \limits _{1 \le i \le m} {1 \over M_i} f_i(x_k) &{} \le &{} h_{k(N)}, \quad k \in {{{\mathcal {A}}}}_0(N). \end{array} \end{aligned}$$

Since $\phi ({{\bar{\lambda }}}^*(N)) \le f^*_0$, this inequality justfies that the point $x^*_N$ is a good approximate solution to primal problem (6.1).

However, note that in our reasonings, we did not assume yet the *existence* of an optimal solution to the dual problem (6.4). It appears that under our assumptions, this may not happen. In this case, since $f_0^*(N)$ can be *significantly smaller* than $f_0^*$, inequality (8.1) cannot justify that vector ${{\bar{\lambda }}}^*(N)$ delivers a good value of the dual objective function.

Let us look at the following example.

### Example 4

Consider the following problem

$$\begin{aligned} \begin{array}{c} \min \limits _{x \in {\mathbb {R}}^2} \Big \{ x^{(2)}:\; 1 -x^{(1)}\le 0, \; \Vert x \Vert _2 \le 1 \Big \}. \end{array} \end{aligned}$$

In this case, ${{{\mathcal {L}}}}(x,\lambda ) = x^{(2)} + \lambda (1 - x^{(1)})$. Hence,

$$\begin{aligned} \begin{array}{rcl} \phi (\lambda )= & {} \min \limits _{\Vert x \Vert _2 \le 1} \mathcal{L}(x,\lambda ) \; = \; \lambda - [1 + \lambda ^2]^{1/2}. \end{array} \end{aligned}$$

Thus, there is no duality gap: $\phi _* {\mathop {=}\limits ^{\textrm{def}}}\sup \limits _{\lambda \ge 0} \phi (\lambda ) = 0$. However, the optimal dual solution $\lambda ^*$ does not exist.

Let us look now at the perturbed feasible set

$$\begin{aligned} \begin{array}{rcl} {{{\mathcal {F}}}}()\epsilon )= & {} \{ x \in {\mathbb {R}}^2: \; \Vert x \Vert _2 \le 1, \; 1 - \epsilon \le x^{(1)} \}, \end{array} \end{aligned}$$

where $\epsilon > 0$ is sufficiently small. Note that it contains a point with the second coordinate equal to $\phi _* - \sqrt{\epsilon (2-\epsilon )}$. This means that the condition (8.1) can guarantee only that

$$\begin{aligned} \begin{array}{rcl} \phi (\lambda ^*(N)\ge & {} \phi _* - \epsilon - \sqrt{\epsilon (2-\epsilon )}. \end{array} \end{aligned}$$

Hence, for dual problems with nonexisting optimal solutions, we can expect a significant drop in the quality of approximation in terms of the function value. $\square$

Thus, in our complexity bounds, we need to take into account the size of optimal dual solution ${{\bar{\lambda }}}^* \in {\mathbb {R}}^m_+$. Let the sequence $\{ x_k \}_{k \ge 0}$ be generated by one of the methods (6.9) or (7.1). Then, for any $k \ge 0$, we have

8.2 $$\begin{aligned} \begin{array}{rl} f_0(x_k) \ge &{} \min \limits _{x \in Q} \Big \{ f_0(x): \; {{\bar{f}}}(x) \le {{\bar{f}}}(x_k) \Big \} = \min \limits _{x \in Q} \max \limits _{{{\bar{\lambda }}} \in {\mathbb {R}}^m_+} \Big \{ f_0(x) + \langle {{\bar{\lambda }}}, {{\bar{f}}}(x) - {{\bar{f}}}(x_k) \rangle \Big \}\\ \\ = &{} \max \limits _{{{\bar{\lambda }}} \in {\mathbb {R}}^m_+} \min \limits _{x \in Q} \Big \{ f_0(x) + \langle {{\bar{\lambda }}}, {{\bar{f}}}(x) - {{\bar{f}}}(x_k) \rangle \Big \} \; = \; \max \limits _{{{\bar{\lambda }}} \in {\mathbb {R}}^m_+} \Big \{ \phi ({{\bar{\lambda }}}) - \langle {{\bar{\lambda }}}, {{\bar{f}}}(x_k) \rangle \Big \}\\ \\ \ge &{} f^*_0 - \langle {{\bar{\lambda }}}^*, {{\bar{f}}}(x_k) \rangle \; \ge \; f^*_0 - \langle {{\bar{\lambda }}}^*, {{\bar{M}}} \rangle \max \limits _{1 \le i \le m} {1 \over M_i} f_i(x_k). \end{array}\nonumber \\ \end{aligned}$$

Thus, we have proved the following theorem.

### Theorem 6

Under conditions of Theorem 4 or 5, for all $N \ge 1 + a(0)$ and all $k \in {{{\mathcal {A}}}}_0(N)$, we have

8.3 $$\begin{aligned} \begin{array}{rcl} f_0^* - \phi ({{\bar{\lambda }}}^*(N))\le & {} (M_0 + \langle {{\bar{\lambda }}}^*, {{\bar{M}}} \rangle ) \, h_{k(N)}. \end{array} \end{aligned}$$

### Proof

Indeed, in view of inequalities (6.12) and (7.3), we have $\max \limits _{1 \le i \le m} {1 \over M_i} f_i(x_k) \le h_{k(N)}$. Thus, we get the bound (8.3) from (8.1) and (8.2). $\square$

Recall that the size of optimal dual multipliers can be bounded by the standard *Slater condition*. Namely, let us assume existence of a point ${{\hat{x}}} \in Q$ such that

8.4 $$\begin{aligned} \begin{array}{rcl} f_i({{\hat{x}}})< & {} 0, \quad i = 1, \dots , m. \end{array} \end{aligned}$$

Then, in accordance to Lemma 3.1.21 in [5], we have

8.5 $$\begin{aligned} \begin{array}{rcl} \langle {{\bar{\lambda }}}^*, - {{\bar{f}}}({{\hat{x}}}) \rangle\le & {} f_0({{\hat{x}}}) - f^*_0. \end{array} \end{aligned}$$

Therefore,

$$\begin{aligned} \begin{array}{rcl} \langle {{\bar{\lambda }}}^*, \bar{M} \rangle &{} = &{} \sum \limits _{i=1}^m \lambda ^*_i(-f_i({{\hat{x}}})) \cdot {M_i \over - f_i({{\hat{x}}})} \; \le \; \langle {{\bar{\lambda }}}^*, - {{\bar{f}}}({{\hat{x}}}) \rangle \max \limits _{1 \le i \le m} {M_i \over - f_i({{\hat{x}}})}\\ \\ &{} {\mathop {\le }\limits ^{(8.5)}} &{} (f_0({{\hat{x}}}) - f^*_0) \max \limits _{1 \le i \le m} {M_i \over - f_i({{\hat{x}}})}. \end{array} \end{aligned}$$

Thus, the Slater condition provides us with the following bound:

8.6 $$\begin{aligned} \begin{array}{rcl} f_0^* - \phi ({{\bar{\lambda }}}^*(N))\le & {} \Big (M_0 + (f_0({{\hat{x}}}) - f^*_0) \max \limits _{1 \le i \le m} {M_i \over - f_i({{\hat{x}}})} \Big ) \, h_{k(N)}, \end{array} \end{aligned}$$

which is valid for all $N \ge 1 + a(0)$. Note that we are able to compute vector ${{\bar{\lambda }}}^*(N)$ without computing values of the dual function $\phi (\cdot )$, which can be very complex. In fact, computational complexity of a single value $\phi (\lambda )$ can be of the same order as the complexity of solving the initial problem (6.1), or even more.

## Subgradient method for unbounded feasible set

In the previous sections, we looked at optimization methods applicable to the bounded sets (see condition (6.3)). If this is not true, the second-order divergence condition (5.6) cannot help, and we need to find another way of justifying efficiency of the subgradient schemes. This is the goal of the current section.

We are still working with the problem (6.1), satisfying condition (6.2). However, the set *Q* is not bounded anymore. Hence, we cannot count on Sion’s theorem (6.4).

In our method, we have a sequence of scaling coefficients $\Gamma = \{ \gamma _k \}_{k \ge 0}$ satisfying condition

9.1 $$\begin{aligned} \begin{array}{rcl} \gamma _{k+1}> & {} \gamma _k \; \ge \; 0, \quad k \ge 0, \end{array} \end{aligned}$$

a tolerance parameter $\epsilon > 0$, and a rough estimate for the distance to the optimum $D_0 > 0$.

For functions $\varphi _{i,y}(\cdot )$ defined by (6.5) with $y \in Q$, denote by $a = a_{i,k}(y)$ the unique solution of the equation

9.2 $$\begin{aligned} \begin{array}{rcl} \varphi _{i,y}\left( {a\over \gamma _{k+1}} \right)= & {} \left( 1 - {\gamma _k \over \gamma _{k+1}} \right) D_0, \quad i = 0, \dots , m. \end{array} \end{aligned}$$

Let us look at the following optimization scheme.9.3It seems that now, the selection of violated constraint by Step 2 looks more natural than in the methods (6.9) and (7.1).

For $x \in Q$, denote $\Delta _k(x) = \gamma _k\Big (\beta _d(x_k,x) - \beta _d(x_0,x)\Big )$. This is a linear function of *x*. In what follows, we assume the Bregman distance is convex with respect to its first argument:

9.4 $$\begin{aligned} \begin{array}{rcl} \beta _d(\alpha u_1 + (1-\alpha )u_2,x) &{} \le &{} \alpha \beta _d(u_1,x) + (1- \alpha ) \beta _d(u_2,x), \\ \\ &{} &{} \forall u_1,u_2, x \in Q, \; \alpha \in [0,1]. \end{array} \end{aligned}$$

This property is not very common. However, it is valid for the following two important examples.

### Example 5

**1.** Let $d(x) = \hbox { }\ {1 \over 2}\Vert x \Vert ^2_B$ ( see (1.5)). Then $\beta _d(x,y) = \hbox { }\ {1 \over 2}\Vert x - y \Vert ^2_B$ and (9.4) holds.

**2.** Let $Q = \{ x \in {\mathbb {R}}^n_+: \; \sum \limits _{i=1}^n x^{(i)} = 1 \}$. Define $d(x) = \sum \limits _{i=1}^n x^{(i)} \ln x^{(i)}$. Then, for $x, y \in Q$, we have $\beta _d(x,y) = \sum \limits _{i=1}^n y^{(i)} \ln {y^{(i)} \over x^{(i)}}$, and (9.4) holds also. $\square$

Now we can prove the following statement.

### Lemma 6

Let the Bergman distance $\beta _d(\cdot ,\cdot )$ satisfy (9.4). Then, for the sequence $\{ x_k \}_{k \ge 0}$ generated by method (9.3), and any $x \in Q$, we have

9.5 $$\begin{aligned} \begin{array}{rcl} \Delta _{k+1}(x)\le & {} \Delta _k(x) + a_k \langle f'_{i_k}(y_k), x - y_k \rangle + (\gamma _{k+1} - \gamma _k) D_0, \quad k \ge 0. \end{array} \end{aligned}$$

### Proof

Note that

$$\begin{aligned} \begin{array}{rcl} \Delta _{k+1}(x) - \Delta _k(x) &{} = &{} \gamma _{k+1} \Big ( \beta _d(x_{k+1},x) - \beta _d(x_0,x) \Big ) - \gamma _{k} \Big ( \beta _d(x_{k},x) - \beta _d(x_0,x) \Big )\\ \\ &{} {\mathop {\le }\limits ^{(9.4)}} &{} \gamma _{k+1} \Big ( \beta _d(x_{k+1},x) - \beta _d(y_k,x) \Big )\\ \\ &{} {\mathop {=}\limits ^{(1.6)}} &{} \gamma _{k+1} \Big ( \langle \nabla d(y_k) - \nabla d(x_{k+1}), x - x_{k+1} \rangle - \beta _d(y_k,x_{k+1} \rangle \Big ). \end{array} \end{aligned}$$

Since the first-order optimality conditions for point $x_{k+1}$ tells us that

$$\begin{aligned} \begin{array}{rcl} \langle a_k f'_{i_k}(y_k) + \gamma _{k+1}(\nabla d(x_{k+1}) - \nabla d(y_k)), x - x_{k+1} \rangle\ge & {} 0, \quad x \in Q, \end{array} \end{aligned}$$

we have

$$\begin{aligned} \begin{array}{rcl} \Delta _{k+1}(x) - \Delta _k(x) &{} \le &{} a_k \langle f'_{i_k}(y_k), x - x_{k+1} \rangle - \gamma _{k+1} \beta _d(y_k, x_{k+1} \rangle \\ \\ &{} {\mathop {=}\limits ^{(6.5)}} &{} a_k \langle f'_{i_k}(y_k), x - y_k \rangle + \gamma _{k+1} \varphi _{i,y_k} \left( {a_k \over \gamma _{k+1}}\right) \\ \\ &{} {\mathop {=}\limits ^{(9.2)}} &{} a_k \langle f'_{i_k}(y_k), x - y_k \rangle + (\gamma _{k+1} - \gamma _k) D_0, \end{array} \end{aligned}$$

and this is inequality (9.5). $\square$

Since $\Delta _0(x) = 0$, we can sum up the inequalities (9.5) for $k = 0, \dots , N-1$ with $N \ge 1$, and get the following consequence:

9.6 $$\begin{aligned} \begin{array}{rcl} \gamma _N \beta _d(x_N, x) + \sum \limits _{k=0}^{N-1} a_k \langle f'_{i_k}(y_k), y_k - x \rangle&{\mathop {\le }\limits ^{(9.5)}}&\gamma _N ( \beta _d(x_0,x) + D_0), \end{array} \end{aligned}$$

which is valid for all $x \in Q$.

In order to approximate the optimal Lagrange multipliers of problem (6.4), we need to introduce the following objects:

9.7 $$\begin{aligned} \begin{array}{rcl} {{{\mathcal {S}}}}_i(N) &{} = &{} \{ k: \; 0 \le k \le N-1, \; i_k = i \}, \quad N \ge 1,\\ \\ {{\hat{\sigma }}}_i(N) &{} = &{} \sum \limits _{k \in {{{\mathcal {S}}}}_i(N)} a_k, \quad i = 0, \dots , m,\\ \\ {{\hat{\lambda }}}_i(N) &{} = &{} {{\hat{\sigma }}}_i(N)/ {{\hat{\sigma }}}_0(N), \quad i = 1, \dots , m. \end{array} \end{aligned}$$

Denote ${{\hat{\lambda }}}(N) = ({{\hat{\lambda }}}_1(N), \dots , \hat{\lambda }_m(N))^T \in {\mathbb {R}}^m_+$ and $f_0^*(N) = \min \limits _{k \in \mathcal{S}_0(N)} f_0(x_k)$. Note that for all $k \in {{{\mathcal {S}}}}_0(N)$ we have

9.8 $$\begin{aligned} \begin{array}{rcl} f_i(x_k)\le & {} \epsilon , \quad k = 1, \dots , m. \end{array} \end{aligned}$$

For our convergence result, we need to assume existence of the optimal solution $x^*$ to problem (6.1). Let us introduce some bound

$$\begin{aligned} \begin{array}{rcl} D\ge & {} \beta _d(x_0,x^*), \end{array} \end{aligned}$$

which is not used in the method (9.3). Denote $Q_D = \{ x \in Q: \; \beta _d(x_0,x) \le D \}$. Clearly, if we replace in problem (6.1) the set *Q* by $Q_D$, then its optimal solution will not be changed. However, now we can correctly define a *restricted* dual function $\phi _D(\lambda ) = \min \limits _{x \in Q_D} {{{\mathcal {L}}}}(x,\lambda )$. Let us prove our main result.

### Theorem 7

Let functional components of problem (6.1) satisfy the following condition:

9.9 $$\begin{aligned} \begin{array}{rcl} \Vert f'_i(x) \Vert _*\le & {} M, \quad i = 0, \dots , m. \end{array} \end{aligned}$$

Then, as far as

9.10 $$\begin{aligned} \begin{array}{rcl} \Sigma _N \; {\mathop {=}\limits ^{\textrm{def}}}\; {1 \over \gamma _N} \sum \limits _{k=0}^{N-1} \Big [\gamma _{k+1}(\gamma _{k+1} - \gamma _k)\Big ]^{1/2}> & {} {\rho _0 + D_0 \over \sqrt{2D_0}} \cdot {M \over \epsilon }, \end{array} \end{aligned}$$

where $\rho _0 = \min \limits _{y \in {{{\mathcal {F}}}}}\beta _d(x_0,y)$, we have ${{{\mathcal {S}}}}_0(N) \ne \emptyset$ and ${{\hat{\sigma }}}_0(N) > 0$. Moreover, if

9.11 $$\begin{aligned} \begin{array}{rcl} \Sigma _N> & {} {D + D_0 \over \sqrt{2D_0}} \cdot {M \over \epsilon }, \end{array} \end{aligned}$$

then

9.12 $$\begin{aligned} \begin{array}{rcl} f^*_0(N) - f^*_0 \; \le \; f^*_0(N) - \phi _D({{\hat{\lambda }}}(N))\le & {} \epsilon . \end{array} \end{aligned}$$

### Proof

Indeed, for any $x \in Q$, we have

$$\begin{aligned} \begin{array}{rcl} \sum \limits _{k=0}^{N-1} a_k \langle f'_{i_k}(y_k), y_k - x \rangle &{} = &{} \sum \limits _{k \in {{{\mathcal {S}}}}_0(N)} a_k \langle f'_0(y_k), y_k - x \rangle + \sum \limits _{i=1}^m \sum \limits _{k \in {{{\mathcal {S}}}}_i(N)} a_k \langle f'_i(y_k), y_k - x \rangle \\ \\ &{} \ge &{} \sum \limits _{k \in {{{\mathcal {S}}}}_0(N)} a_k [f_0(y_k) - f_0(x)] + \sum \limits _{i=1}^m \sum \limits _{k \in {{{\mathcal {S}}}}_i(N)} a_k [f_i(y_k) - f_i(x)]\\ \\ &{} \ge &{} {{\hat{\sigma }}}_0(N) [f^*_0(N) - f_0(x)] + \sum \limits _{i=1}^m {{\hat{\sigma }}}_i(N)[\epsilon - f_i(x)]. \end{array} \end{aligned}$$

Hence, in view of inequality (9.6), we get the following bound

$$\begin{aligned} \begin{array}{rcl} \hat{\sigma }_0(N) [f^*_0(N) - f_0(x)] + \sum \limits _{i=1}^m \hat{\sigma }_i(N)[\epsilon - f_i(x)]\le & {} \gamma _N(\beta _d(x_0,x) + D_0). \end{array} \end{aligned}$$

Note that ${\hat{\sigma }}_0(N) + \sum \limits _{i=1}^m {{\hat{\sigma }}}_i(N) = \sum \limits _{k=0}^{N-1} a_k$. Therefore, this inequality can be rewritten as follows:

9.13 $$\begin{aligned} \begin{array}{rcl} \gamma _N(\beta _d(x_0,x) + D_0)\ge & {} {{\hat{\sigma }}}_0(N) [ f^*_0(N)- f_0(x) - \epsilon ] + \epsilon \sum \limits _{k=0}^{N-1} a_k - \sum \limits _{i=1}^m {{\hat{\sigma }}}_i(N) f_i(x). \end{array}\nonumber \\ \end{aligned}$$

In view of condition (9.2), for $T_i = T_{i,y_k}\left( {a_{i,k}(y_k) \over \gamma _{k+1}} \right)$, we have

$$\begin{aligned} a_{i,k}(y_k) \langle f'_i(y_k), y_k - T_i \rangle \;{} & {} = \; \gamma _{k+1} \beta _d(y_k,T_i) + (\gamma _{k+1} - \gamma _k) D_0\\{} & {} {\mathop {\ge }\limits ^{(1.8)}} \hbox { }\ {1 \over 2}\gamma _{k+1} \Vert T_i - y_k \Vert ^2 + (\gamma _{k+1} - \gamma _k) D_0 \; \\{} & {} \ge \; \Big [ 2 \gamma _{k+1} (\gamma _{k+1} - \gamma _k) D_0 \Big ]^{1/2} \Vert T_i - y_k \Vert . \end{aligned}$$

Thus, $M a_k(y_k) {\mathop {\ge }\limits ^{(9.9)}} \Big [ 2 \gamma _{k+1} (\gamma _{k+1} - \gamma _k) D_0 \Big ]^{1/2}$, and we conclude that

9.14 $$\begin{aligned} \begin{array}{rcl} \sum \limits _{k=0}^{N-1} a_k\ge & {} {1 \over M}\sqrt{2D_0} \gamma _N \Sigma _N. \end{array} \end{aligned}$$

Let us assume that ${{{\mathcal {S}}}}_0(N) = \emptyset$. Then $\hat{\sigma }_0(N) = 0$ and for $x = \arg \min \limits _{y \in {{{\mathcal {F}}}}} \beta _d(x_0,y)$ inequality (9.13) leads to the following relation:

$$\begin{aligned} \begin{array}{rcl} \gamma _N( \rho _0 + D_0)&{\mathop {\ge }\limits ^{(9.14)}}&{\epsilon \over M} \sqrt{2D_0} \gamma _N \Sigma _N. \end{array} \end{aligned}$$

However, this cannot happen in view of condition (9.10). Hence, inequality (9.10) implies ${{{\mathcal {S}}}}_0(N) \ne \emptyset$ and ${{\hat{\sigma }}}_0(N) > 0$. In this case, inequality (9.13) can be rewritten as follows:

$$\begin{aligned} \begin{array}{rcl} \beta _d(x_0,x) + D_0&{\mathop {\ge }\limits ^{(9.14)}}&{1 \over \gamma _N}\hat{\sigma }_0(N) [ f^*_0(N)- {{{\mathcal {L}}}}(x, {{\hat{\lambda }}}(N)) - \epsilon ] + {\epsilon \over M}\sqrt{2D_0} \Sigma _N. \end{array} \end{aligned}$$

Maximizing the right-hand side of this inequality in $x \in Q_d$, we get

$$\begin{aligned} \begin{array}{rcl} D + D_0\ge & {} {1 \over \gamma _N}{{\hat{\sigma }}}_0(N) [ f^*_0(N)- \phi _D({{\hat{\lambda }}}(N)) - \epsilon ] + {\epsilon \over M}\sqrt{2D_0} \Sigma _N. \end{array} \end{aligned}$$

Hence, inequality (9.11) implies (9.12). $\square$

Thus, for the fast rate of convergence of method (9.3), we need to ensure a fast growth of the valies $\Sigma _N$. Let us choose

9.15 $$\begin{aligned} \begin{array}{rcl} \gamma _k= & {} \sqrt{k}, \quad k \ge 0. \end{array} \end{aligned}$$

Then $$\gamma _{k+1}(\gamma _{k+1} - \gamma _k) = {\sqrt{k+1} \over \sqrt{k+1} + \sqrt{k}} \ge \hbox { }\ {1 \over 2}$$. Hence,

9.16 $$\begin{aligned} \begin{array}{rcl} \Sigma _N\ge & {} {1 \over \sqrt{N}} \cdot {N \over \sqrt{2}} \; = \; \sqrt{N \over 2}. \end{array} \end{aligned}$$

In this case, inequality (9.12) can be ensured in $O(\epsilon ^{-2})$ iterations.

Note that method (9.3) is quite different from the existing optimization schemes. Let us write down how it looks like in the case $Q = {\mathbb {E}}$, $d(x) = \hbox { }\ {1 \over 2}\Vert x \Vert ^2_B$, and the parameter choice (9.15). In this case, the Eq. (9.2) can be written as follows:

$$\begin{aligned} \begin{array}{rcl} {a^2 \over 2 \gamma _{k+1}^2} \Vert f'_i(y_k) \Vert ^2= & {} {\gamma _{k+1} - \gamma _k \over \gamma _{k+1}} D_0. \end{array} \end{aligned}$$

Thus, $a_k = \sqrt{2 \gamma _{k+1}(\gamma _{k+1} - \gamma _k) D_0 } \cdot {1 \over \Vert f'_{i_k}(y_k) \Vert ^*_B} \approx {\sqrt{D_0} \over \Vert f'_{i_k}(y_k) \Vert ^*_B}$, and the method looks as follows:9.17As compared with other known switching primal-dual schemes (e.g. Section 3.2.5 in [5]), in method (9.17), we apply variable step sizes, which allow faster movements in the beginning of the process.

## Conclusions

In this paper, we presented several new *primal* subgradient methods with a better control of step sizes. Our main observation is that for constrained minimization problems, in a proper definition of the actual size of subgradients of convex function, we must take into account the position of the test point with respect to the boundary of the feasible set. In the simplest variant, this can be done by ensuring the size of the proximal step to be equal to a prescribed control parameter.

In this way, we develop new methods for solving quasi-convex problems (Sect. 2) and show that their convergence rate automatically adjust the favorable local structure of the optimal solution. After that, in Sects. 3 and 4, we analyze new schemes for minimizing max-type convex functions and functions with smooth strongly convex components. Our main theoretical improvement there is the scheme with the linear rate of convergence for the problem, where the classical step-size strategy ensures only a sublinear rate.

The remaining part of the paper is devoted to problems with non-trivial functional constraints. In Sect. 5, we present a method for solving problems with single constraint in the composite form (it seems that this formulation is new). For treating this kind of problems, we introduce a new condition of *diverging series of squares* of the control step-size parameters and define *divergence delay*, which shows how quickly the method can eliminate the past. Our new method can automatically adjust to the best Hölder class containing the functional components.

In the last three sections of the paper, we present methods, which can efficiently approximate optimal Lagrange Multipliers by a simple switching strategies. All these schemes ensure the optimal rate of convergence. They differ one from another by involvement of Slater condition into the final efficiency bound and by the way they treat unbounded feasible sets.

Our new technique is based on ability to solve some auxiliary univariate problems, related to the prox-type operations. However, for the simple feasible sets, these problems are easy. As a benefit, we get new methods which can adjust to the problem structure and move with much longer steps, especially in the beginning of the process.
